# Non-Hydroxamate Zinc-Binding Groups as Warheads for Histone Deacetylases

**DOI:** 10.3390/molecules26175151

**Published:** 2021-08-25

**Authors:** Anton Frühauf, Franz-Josef Meyer-Almes

**Affiliations:** Department of Chemical Engineering and Biotechnology, University of Applied Sciences Darmstadt, Haardtring 100, 64295 Darmstadt, Germany; anton.fruehauf@h-da.de

**Keywords:** histone deacetylase (HDAC), histone deacetylase inhibitors (HDACis), zinc-binding group (ZBG), non-hydroxamate

## Abstract

Histone deacetylases (HDACs) remove acetyl groups from acetylated lysine residues and have a large variety of substrates and interaction partners. Therefore, it is not surprising that HDACs are involved in many diseases. Most inhibitors of zinc-dependent HDACs (HDACis) including approved drugs contain a hydroxamate as a zinc-binding group (ZBG), which is by far the biggest contributor to affinity, while chemical variation of the residual molecule is exploited to create more or less selectivity against HDAC isozymes or other metalloproteins. Hydroxamates have a propensity for nonspecificity and have recently come under considerable suspicion because of potential mutagenicity. Therefore, there are significant concerns when applying hydroxamate-containing compounds as therapeutics in chronic diseases beyond oncology due to unwanted toxic side effects. In the last years, several alternative ZBGs have been developed, which can replace the critical hydroxamate group in HDACis, while preserving high potency. Moreover, these compounds can be developed into highly selective inhibitors. This review aims at providing an overview of the progress in the field of non-hydroxamic HDACis in the time period from 2015 to present. Formally, ZBGs are clustered according to their binding mode and structural similarity to provide qualitative assessments and predictions based on available structural information.

## 1. Introduction

**Figure 1:**
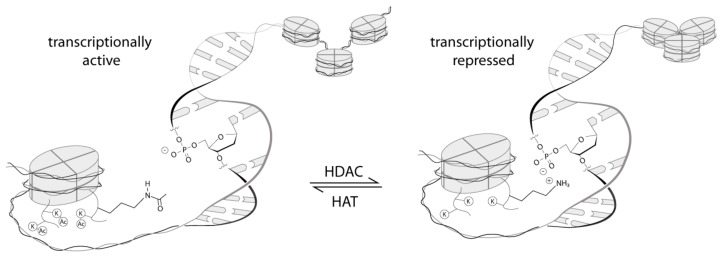
Simplistic illustration of interactions between histone tail lysine residues and the DNA backbone, as well as its effects on chromatin condensation and, finally, transcriptional accessibility.

Histone deacetylases (HDACs) belong to the machinery of the epigenetic apparatus and play a crucial role in chromatin remodeling by altering posttranscriptional modifications and controlling gene transcription, which is maladjusted in various cancers, leading to altered transcription of onco- and tumor suppressor genes [1]. One of the most common post-translational modifications is the (de)acetylation of histone residues by HDAC or histone acetyl transferase (HAT), its antagonist [2]. The reversible removal of acetyl groups from ε-amino lysine residues of histone tails establishes ionic contacts between the negatively charged DNA and the positively charged lysine residue which causes the chromatin to condense, whereas the addition of acetyl groups by HAT disables this interaction, causes the chromatin to loosen up, and permits gene transcription [3] (Figure 1). Histone deacetylase inhibitors (HDACis) exhibit a wide range of cytotoxic effects and facilitate hyperacetylation, thus permitting the transcription and activation of genes such as p53, p21^Waf/Cip1^, Gadd 45, FAS, and caspase-3, which are associated with cell-cycle arrest, differentiation, and apoptosis [4,5,6,7,8,9]. This possible therapeutic potential of HDACs has attracted increasing attention not only for the specified reason but also because of the ability to deacetylate non-histone proteins that are commonly involved in cancer metabolism [10,11,12,13,14].

**Figure 2:**
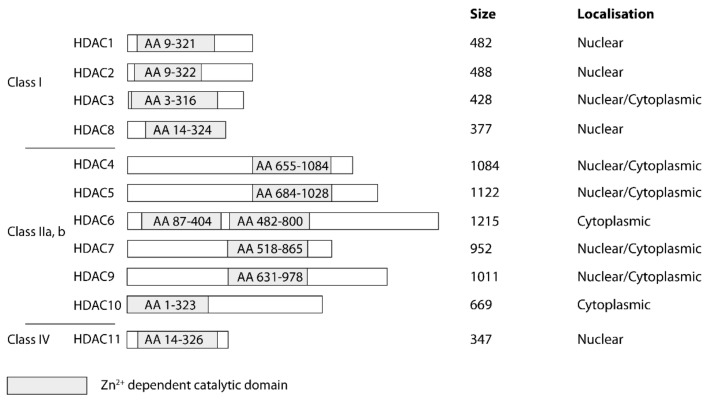
Schematic classification of HDACs with indicated catalytic domain size, protein length, and localization.

Generally, two varieties of enzymes exist among the 18 human HDACs, which adopt either a zinc-dependent arginase fold or an NAD^+^-dependent Rossmann fold [15,16]. Altogether, they can be grouped into four classes (classes I–IV). Classes I, II, and IV are zinc-dependent enzymes, whereas the seven members of class III are called sirtuins (SIRT1–7) and are not considered in this review (Figure 2). Class I consists of HDAC1–3 and HDAC8, which are predominantly expressed in the nucleus. Class IIa/b HDACs include HDAC4–7 and HDAC9–10, which can shuttle between the nucleus and cytoplasm according to their phosphorylation state and their localization domains. HDAC11 is the only member of class IV and is localized in the nucleus [17,18].

**Figure 3:**
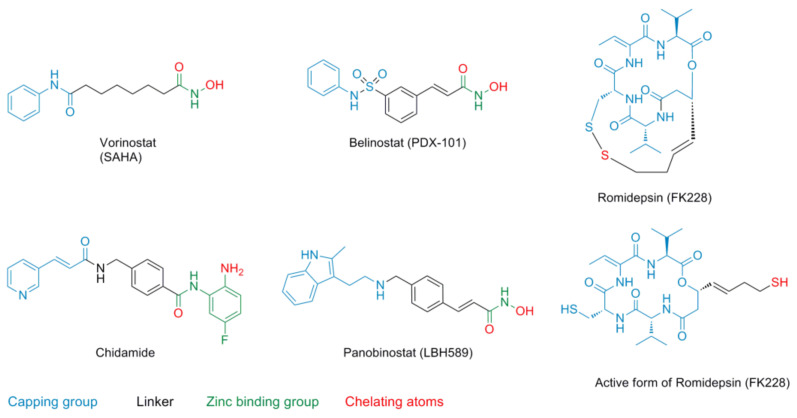
Representation of FDA-approved HDAC inhibitors with indicated structural features. The cap group is depicted in blue and is connected via the carbon linker (black) to the ZBG (green), of which the chelating atoms are highlighted in red.

In addition to being interesting targets for cancer, some HDACs are associated with neuronal plasticity and are involved in diseases such as peripheral nerve disorder, Parkinson’s disease, or Huntington’s disease [19,20]. Today, several HDACis are approved by the Food and Drug Administration of the USA and China (Figure 3). Treatment of cutaneous T-cell lymphoma with vorinostat (SAHA) [21], romidepsin (FK228) [22], and belinostat (PDX-101) [23] is approved on the US market, while panbinostat (LBH-589) [24] is approved for the treatment of multiple myeloma. Recently, China approved chidamide [25] for the treatment of peripheral T-cell lymphoma.

HDACis commonly consist of a cap group, which is mostly used to enhance selectivity, a linker with a suitable length and steric demand, and a zinc-binding group (ZBG) for chelation. 

**Figure 4:**
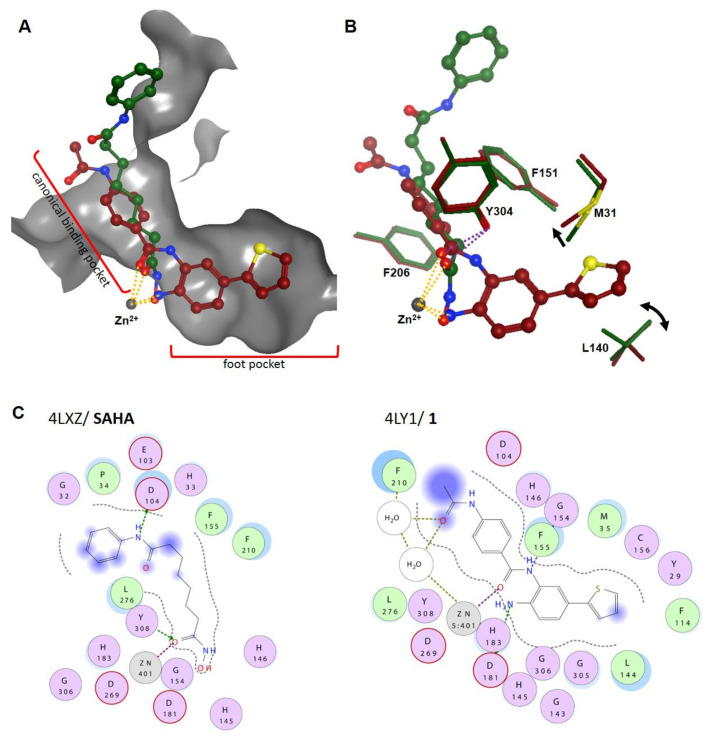
Structural overlay of HDAC2 complexed with SAHA (PDB ID: 4LXZ) and the benzamide compound **1** (PDB ID: 4LY1; Lauffer et al. [30]). (**A**) Clipped binding pocket indicating the canonical binding pocket for substrate recognition and the widened foot pocket, which is also referred to as the acetate release channel. The zinc ion is shown as a gray sphere, and the clipped surface of the pocket is colored in gray. Metal bonds are shown as dotted orange lines. (**B**) L140 changes rotamers and M31 shifts to the side to open the foot pocket for the thiophenyl moiety. Hydrogen bonds are indicated as magenta dotted lines. (**C**) The 2D molecular interactions of SAHA and **1** within the canonical binding site of HDAC2. Side-chain hydrogen bonds are indicated by dotted green lines, whereas backbone hydrogen bonds are indicated as blue dotted lines. Metal complexation is shown using dotted magenta lines, solvent contacts are shown as dotted ochre lines, and exposure is illustrated with blue shading. Residues are numbered according to the crystal structure; for canonical numbering, refer to (**A**) and (**B**).

The crystal structure of histone deacetylase-like protein (HDLP) with SAHA was the first solved structure, which represents the typical binding mode and helped to understand the three-dimensional structure and the catalytic mechanism of HDACs, whose details are still under debate [26,27,28,29]. Since then, a variety of human HDAC structures have been solved together with several HDACis [30,31]. Below, the crystal structures of HDAC2 with SAHA (PDB ID: 4LXZ) and amino benzamide derivative **1** (PDB ID: 4LY1) are described (Figure 4A,B). Both ZBGs coordinate the zinc ion in a bidentate fashion, whereby SAHA chelates through the OH (2.0 Å) and the CO (2.3 Å) groups and **1** chelates via the NH_2_ (1.9 Å) and CO (2.6 Å) moieties with distances indicated in brackets. Additionally, SAHA forms hydrogen bonds with its OH group and its NHCO group to two His and one Tyr residue (H141, H142, and Y304). Equivalent coordination of the zinc ion is observed for amino benzamides, which in general exhibit a much longer residence time, i.e., the reciprocal of the dissociation rate, compared to hydroxamate HDACi [30]. Additionally, the thiophenyl moiety of compound **1** protrudes into the foot pocket of HDAC2 and interacts with M31 and L140, thereby introducing selectivity for this HDAC isozyme. The linker regions of SAHA and compound **1** pass through the channel made up of the hydrophobic residues L272, F151, G150, and F206, and they project their cap groups on the surface of HDAC2. The surface residues are generally the least conserved among the HDAC family. SAHA interacts with HDAC2 surface residue D100 and a water molecule via hydrogen bond interactions. Unlike SAHA, compound **1** has just a minor extension into the solvent-accessible area and forms hydrogen bonds with its amide group to two adjacent water molecules in proximity to D100 (Figure 4C).

To date, most of the studied HDACis have used a hydroxamic acid as ZBG, which is generally accompanied by poor selectivity, toxicity, and bad pharmacokinetic properties [32,33]. Therefore, therapeutical applications outside of cancer treatment are viewed critically without potent and selective active substances containing non-hydroxamate ZBGs. 

For a better overall understanding, it is beneficial to evaluate the bivalent zinc cation. Zinc ions are among the most abundant and important ions in metallo-enzymes and are unequally distributed among cellular compartments, with the cytosol and the nucleus being the most zinc ion-enriched [34]. Biochemical functions of enzymes depend to a great extent on the coordinated metal ion. With the zinc ion having an [Ar]*d*10 configuration, it is redox-inactive compared to lighter transition metals. The zinc ion is a borderline acid in the concept of HSAB, making it a suitable coordination partner for soft and hard bases, diversifying and adjusting its properties for different functions [35,36]. Zinc can seemingly adopt two states in HDACs, a tetrahedral or penta-coordinated trigonal bipyramidal state, which are dependent on the occupation of the binding site and are representative of the possible mono- or bidentate chelation with ZBGs [35,37,38,39].

Several ZBGs show preference towards different isoforms and can be used to exploit isoform selectivity, minimize off-target effects, circumvent toxic metabolites, and enhance pharmacokinetic properties. Hydroxamates represent the most widespread class of HDACis. Although very potent, the mutagenic potential of this class of active substances was validated in the early 1980s. All hydroxamate HDACis exhibit a positive Ames test and cause anomalies in rodent cells [40]. Poor pharmacokinetic properties such as fast clearance also handicap clinical use, but they are tolerated in the case of a life-threatening disease [33]. Non-hydroxamate ZBGs are usually less potent than hydroxamates, but they can exhibit greater selectivity among isoforms and are potentially less toxic, which might implicate long-term therapeutic use for other non-life-threatening diseases [41]. The aspect of isozyme selectivity was treated in great detail in the excellent recent reviews by Ho et al. [10] and Melesina et al. [42], which are recommended for further reading. In this review, we address the progress in the field of non-hydroxamic HDACis in the time period from 2015 to present. ZBGs are clustered according to their binding mode and structural similarity to provide qualitative assessments and predictions based on available structural information.

## 2. Assays to Determine HDACi Activity

The activity of putative HDACis is usually assessed using a cascade of in vitro and in vivo assays. To critically evaluate data from the literature, it is useful to know the principles behind the applied assays, and the meaning of the obtained results. Therefore, the most widely used assays are briefly described below. First-line assays are usually enzyme activity assays, which measure the inhibitory effect of compounds on the HDAC isozyme of interest by using chromogenic substrates. Such assays can be used in high-throughput campaigns, because they have a homogeneous format, can be measured in parallel on microtiter plates, and require only little amounts of material. Furthermore, dose–response curves can be obtained easily to determine IC_50_ values, which are most frequently used to compare the activity of compounds. Mostly, a deacetylation reaction is coupled to a second detection reaction, where the optical signal, very often luminescence, is developed. In our laboratory, we use a fluorogenic end-point assay, which was described in detail by Meyners et al. [43]. Usually, purified recombinant HDACs are used to enable the assessment of the inhibitory effect of compounds against a specific isozyme. However, some studies use a HeLa nuclear extract as the source of HDACs. In this case, only the overall effect of compounds on a rather undefined mixture of different HDACs is measured. Isozyme specificity cannot be assessed. Despite the widespread use of IC_50_ values, their benefit for the optimization of lead compounds by medicinal chemistry is limited, because this quantity depends strongly on the assay condition and particularly on the substrate concentration. This fact explains the observed large variability of IC_50_ values for the same compounds across different laboratories. If the inhibition mode, for example, competitive inhibition, is known, the binding constant can be calculated from an IC_50_ value using the equations by Cheng and Prusoff [44], which is much better suited for comparing the affinity of active substances between different laboratories. Binding assays are alternative approaches to directly determine binding constants. Several types of binding assays have been developed for different classes of zinc-dependent HDACs. Typical readouts of HDAC binding assays include Förster resonance energy transfer (FRET) [45], time-resolved FRET (TR-FRET), bioluminescence resonance energy transfer (BRET) [46], fluorescence lifetime [47], and fluorescence polarization (FP) [48]. Another method to determine the binding constants of HDAC inhibitors is differential scanning fluorimetry (DSF), also termed thermal shift. This method uses a dye, e.g., SYPRO Orange, which becomes severalfold more fluorescent upon binding to exposed hydrophobic regions of a protein. By heating HDACs in the absence or presence of a ligand, the dye binds to the increasingly exposed hydrophobic residues of amino acids, allowing for continuous monitoring of protein unfolding. Because ligands that bind to HDACs typically stabilize the native protein, this leads to a shift in the midpoint of the unfolding transition, also called the melting point. Binding constants can be calculated from the dependence of the melting point on the ligand concentration [49]. If the functionality of HDACis is shown in biochemical assays, the next step is the demonstration of intracellular target engagement. For this purpose, a BRET assay could be applied, where a fluorescent probe binds to an intracellular HDAC–luciferase fusion protein [50]. After addition of a suitable luciferase substrate, a positive BRET signal is generated. If an active substance penetrates the cell membrane and competes with the probe, a decreasing BRET signal indicates the intracellular interaction between compound and HDAC target. Alternatively, the acetylation status of specific intracellular substrates of a particular HDAC isozyme can be determined to conclude on the intracellular efficacy of an active compound. The amount of acetylated protein substrates is usually determined by using specific antibodies in a quantitative Western blot. Typical substrates of interest are acetylated histones, which are preferred substrates of HDACs 1, 2, and 3, acetylated α-tubulin, a specific substrate for HDAC6 [51], and acetylated SMC3, which is a bona fide substrate of HDAC8 [52]. Since HDACs are predominantly considered as cancer targets, it is of interest to measure the cytotoxic effect of active substances in proliferation assays. A very common assay is the colorimetric MTT assay, which assesses cell metabolic activity. In the next phase, the in vivo efficacy of compounds has to be shown in xenograft hematological or solid tumor models. It is important to note that enzyme activity and biochemical binding assays, as well as Western blots of acetylated HDAC substrates, indicate the molecular interaction between a compound and an HDAC enzyme, while proliferation assays and in vivo xenograft tumor models demonstrate the intended biological outcome of HDAC inhibition. Therefore, it is possible, that compounds are more active in a proliferation assay and have no or little activity in an HDAC activity assay due to nonspecific cellular toxicity.

## 3. Classic Benzamide Warheads

**Figure 5:**
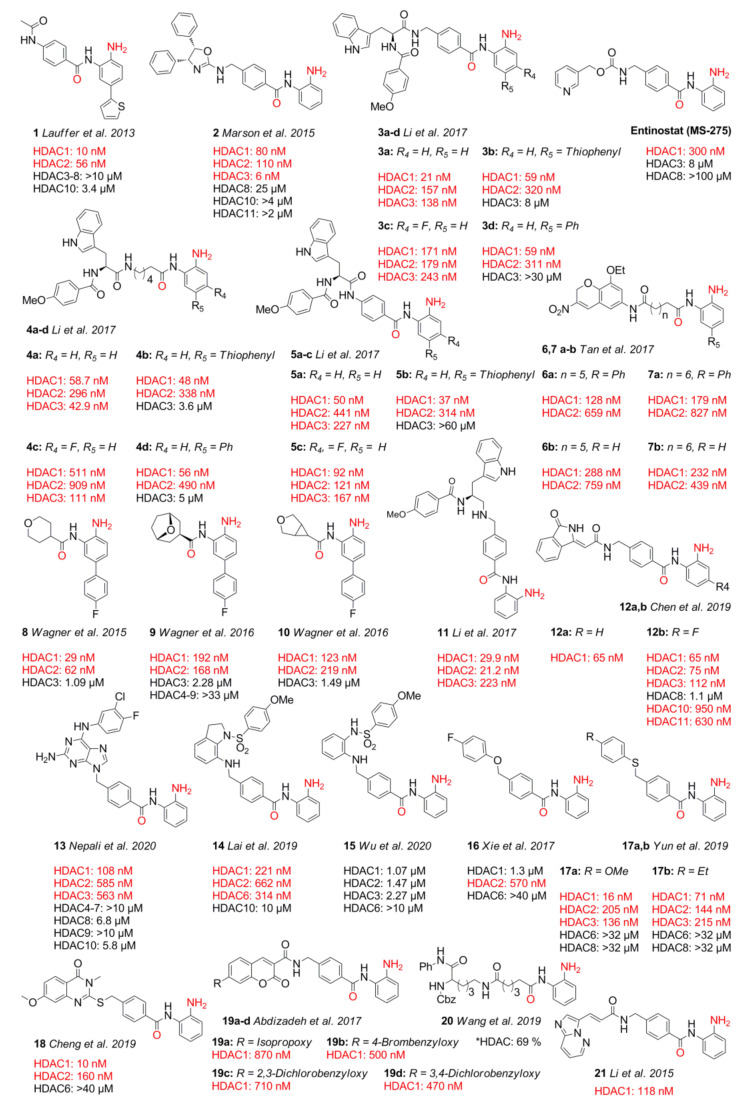
A selection of the latest prepared amino benzamides. IC_50_ values against HDAC isoforms are listed wherever possible. Chelating atoms in compounds and activities below 1 µM are highlighted in red. * Inhibitory activity is given as the percentage inhibition of HDACs in HeLa nuclear extract as an HDAC source in the presence of **20** (100 µM).

**Figure 6:**
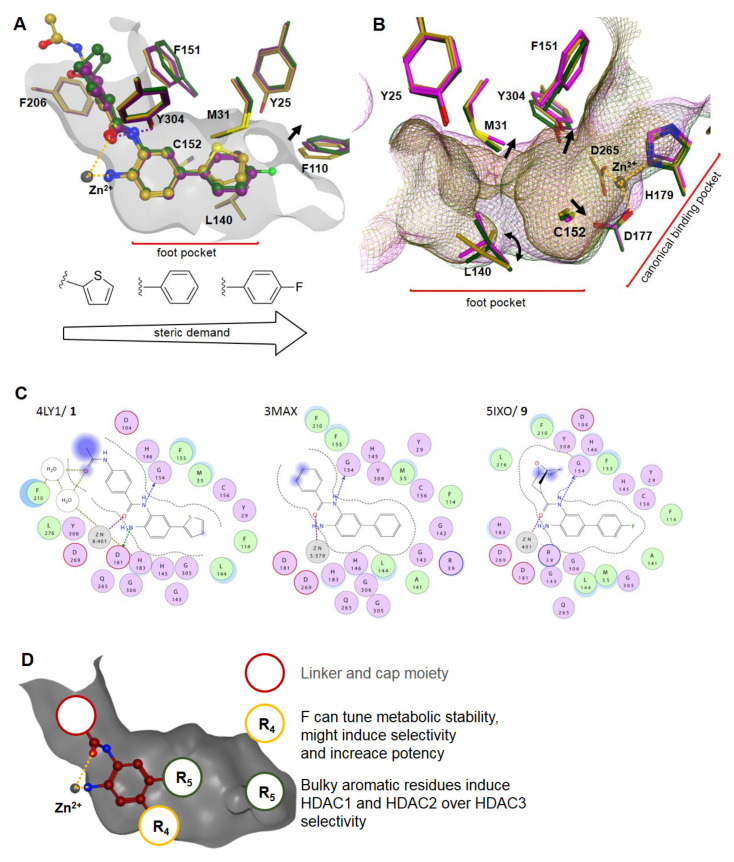
(**A**) Overlay of HDAC2 (PDB ID: 4LY1/ochre, 3MAX/magenta, and 5IX0/green). Three ligands with aryl substitutions indicated below protrude into the foot pocket and displace the F110 residue with increasing steric demand as indicated by the arrow. (**B**) Overlay of HDAC1–3 (PDB ID: HDAC1, 4BKX/magenta; HDAC2, 7LTK/ochre; HDAC3, 4A69/green) and visualization of the surface area around the foot pocket. The surfaces of the foot pocket of HDAC1 (magenta), HDAC2 (ochre), and HDAC3 (green) are shown as mesh. Residues are numbered according to HDAC2 (PDB ID: 7LTK). M31, Y304, and C152 undergo displacement as indicated by the arrows, successively widening the foot pocket from HDAC1 to HDAC3. Rotamers for L140 are indicated with curly arrows and illustrate a gatekeeping property. Metal bonds to zinc-binding residues D265, H179, and D177 are illustrated as orange dotted lines. (**C**) 2D ligand interactions of (**A**) overlaid HDAC2 crystal structures with PDB IDs 4LY1, 3MAX, and 5IXO. (**D**) Schematic SAR of amino benzamide warheads with indicated spheres as placeholders for substituents within the clipped binding pocket of HDAC2 (PDB ID: 4LY1). Metal bonds are indicated as orange dotted lines.

Amino benzamides are a predominant class of HDACi (Figure 5) including the clinically approved drug chidamide (Figure 3) as a representative. Amino benzamides were first reported in 1999 with in vivo antitumor activity and no severe side effects in animal models [53,54]. It was directly evident that the *ortho* amino function is crucial for inhibitory activity [53]. In general, amino benzamides exhibit IC_50_ values in the nM to µM range [55,56,57] with emphasis on compound **2**, exhibiting the lowest IC_50_ value of 6 nM [58]. These warheads are HDAC class I selective and show slow but tight binding properties [30]. The slow binding needs to be accounted for in experiments and might be partially explained by the intramolecular hydrogen bond between the *ortho* amino function and the amide carbonyl group [17]. In order to chelate the zinc ion, this H-bond needs to be broken, which is accompanied by an energetic solvation penalty [17,59]. Common on rates are in the range of 2 × 10^4^ M^−1^⋅min^−1^, and residence times range up to 20 h [30]. Exemplary pharmacokinetic properties for entinostat (MS-275) show a maximum concentration C_max_, after drug administration at the time T_max_, which is in the range of 0.25–2 h. Further low oral clearance (CL/F) and half-life times T_1/2_ of 52 h add to the good absorption, distribution, metabolism, excretion, and toxicity (ADMET) properties [60]. Aromatic amines may, in general, be mutagenic [61,62]. Additionally, amino benzamides seem to have a bias to undergo efficient cyclization under acidic conditions to yield an inactive benzimidazole product [63]. Clinical data on amino benzamide toxicity suggest target- instead of chemical-related side effects, according to observed clinical symptoms shared across different ZBGs [64,65,66,67,68,69,70,71,72,73,74,75]. Until now co-crystallization of amino benzamides has only been successful with HDAC2 (PDB IDs: 4LY1, 3MAX, 5IWG, and 5IX0) [76,77,78]. The amino benzamide ZBG generally chelates the zinc ion via the *ortho* amino group and the amide carbonyl, and it occupies the foot pocket with the phenyl moiety. Mean distances between the respective heteroatom and the zinc ion in the mentioned crystal structures were measured to be in the range of 2.1 Å and 2.46 Å for the amine and carbonyl moieties, respectively. As expected, the amine anchor shows a threefold lower deviation in distance across compared crystal structures in contrast to the to the second heteroatom with a wide range of distances between 2.5 Å and 2.7 Å. Interactions generated by the cap and linker group are of diverse origin and tune the selectivity, potency, pharmacokinetic properties, and toxicity, contributing to the overall pharmacological performance of the inhibitor. The structure–activity relationship (SAR) below is suggested for classic amino benzamide warheads on the basis of the work of Li et al. [79], Tan et al. [80], Chen et al. [81], Bressi et al. [77], and Lauffer et al. [30] (Figure 6).

As depicted in Figure 6A, the thiophenyl moiety protrudes into the foot pocket of HDAC2, providing HDAC1 and HDAC2 selectivity over other isoforms. This foot pocket consists of the residues G301, R35, C151, Y25, F110, P33, I36, A137, G138, R35, Y304, L140, and M31, whereby the latter two undergo a minor rotation and displacement, enlarging the foot pocket for the thiophenyl moiety [30]. Notably, a bulky substituent in R_5_ is accompanied by displacement of F140 at the end of the foot pocket. The displacement correlates with substituent size (Figure 6A) and induces selectivity, as shown by compounds **3–10**, compared to smaller residues or non-substituted compounds such as **11**. Visualization of HDAC1–3 (Figure 6B) shows a displacement of the residues Y304, M31, C152, and L140, which induce pocket expansion for HDAC1 and provide a more beneficial environment for R_5_-substituted HDACis, which is in accordance with IC_50_ data. Exchanging the thiophenyl for a phenyl moiety does not affect HDAC1 and HDAC2 properties but significantly decreases HDAC3 inhibitory activity, leading to increased HDAC1 and HDAC2 selectivity for compounds **3d** and **4d**. A fluorine substitution at position R_4_ (Figure 6D) usually retains or decreases overall potency, as exemplified by **3c–5c** and **12b**. In the case of **4c**, it provides reversed selectivity toward HDAC3. Sterically more demanding groups in R_4_ such as chlorine or methyl are incompatible, leading to a substantial loss of potency.

In 2017, Li et al. [79] synthesized a new series of amino benzamide inhibitors with only slightly decreased activity compared to the hydroxamic acid group used in earlier studies [82]. Installation of several linking units taken from compounds in clinical phase I–III stages yielded compounds that showed HDAC1 and HDAC3 selectivity, of which **3a** exhibited oral in vivo activity against xenograft hematological and solid tumor models with no obvious toxicity. Compounds **3–5** and **11** were potent and inhibited HDAC1–HDAC3 with exemplary IC_50_ values of 58.7 nM (HDAC1), 296 nM (HDAC2), and 42.9 nM (HDAC3) for **4a**, as well as 29.9 nM (HDAC1), 21.2 nM (HDAC2), and 223 nM (HDAC3) for **11**. In proliferation assays, lead compound **3a** was comparably potent to entinostat and exhibited IC_50_ values of 3.02 µM (Hela), 980 nM (K562), 1.10 µM (U937), 2.23 µM (U266), and 4.23 µM (HCT116) and was more potent against K562 and U266 cell lines than entinostat. Further modification of the ZBG of **3–5** yielded derivatives **b–d**. Tan et al. [80] prepared several ZBGs with 3-nitro-2*H*-chromene derivatives as cap moieties. Amino benzamide compounds **6a** and **7a** preferentially inhibited HDAC1 over HDAC2 with IC_50_ values of 128 nM and 179 nM for HDAC1 and 659 nM and 827 nM for HDAC2, being more potent than the reference compound entinostat. Cell-based studies also showed stronger inhibitory activities, being about twofold more effective compared to the reference compounds with GI_50_ values in the range from 2.00 µM to 19.79 µM toward K562, A549, MCF-7, PC-3, and HeLa cell lines. Studies by Wagner et al. [83] led to compound **8**, which was kinetically selective toward HDAC2. In 2016, a new series was synthesized and further investigated, leading to compounds **9** and **10** [78]. Compound **8** exhibited inhibitory activity of 29 nM (HDAC1), 62 nM (HDAC2), and 1.09 µM (HDAC3) and kinetic selectivity over HDAC2 with a residence time of 143 min, as well as k_on_ and k_off_ values of 0.014 min^−1^⋅µM^−1^ and 0.0049 min^−1^ [83]. Further compounds **9** and **10** exhibited IC_50_ values of 192 nM and 123 nM for HDAC1, 168 nM and 219 nM for HDAC2, and 2.28 µM and 1.49 µM for HDAC3 [78]. The kinetic selectivity can be highlighted, which starts to be effective only after the first hour of incubation as the slower off-rate toward HDAC2 is gaining more impact. A study performed by Chen et al. [81] evaluated an isoindolinone cap with different ZBGs. It was found that compounds **12a** and **12b** inhibit HDAC1 with 65 nM and show half-times of 292 min in human liver microsome (HLM) compared to chidamide with a half-life of 276 min. Evaluation of **12b** against several cancer cell lines showed IC_50_ values of 373 nM (HL-60), 193 nM (K562), 432 nM (HCT116), and 14.56 µM (MCF-7) compared to chidamide 1.97 µM (HL-60), 747 nM (K562), 1.09 µM (HCT116), and 29.07 µM (MCF-7). Work by Nepali et al. [84] in 2020 aimed at preventing the formation of toxic metabolites originating from carbamate and acrylamide moieties frequently encountered in HDACi with examples such as entinostat and chidamide [84]. Replacement of these with purine isoster moieties as cap groups yielded compound **13**, which was most selective for HDAC1, exhibiting an IC_50_ value of 108 nM. Cell evaluation against HDACi-resistant and -sensitive gastric cell lines YCC3/7 and YCC11 revealed IC_50_ values of 4.79 µM and 4.77 µM, respectively. In 2019, Lai et al. [85] worked on dual inhibitors composed of a microtubule and HDAC-binding agent, in order to overcome the inactivation seen with microtubule drugs. Compound **14** could inhibit HDAC isoforms with an IC_50_ of 221 nM (HDAC1), 662 nM (HDAC2), and 314 nM (HDAC6). Generated in vitro and cell data with multidrug-resistant (MDR) cells showed improved potencies of **14** compared to the HDACi reference entinostat and a microtubule-binding agent (MTA) reference, incorporating colchicine and stilbene motifs. Compound **14** exhibited IC_50_ values in the range of 49–64 nM against KB cell lines and significant in vivo efficacy in the human non-small-cell lung cancer A549 xenograft model, as well as the B-cell lymphoma BJABB xenograft tumor model. Further work by Wu et al. [86] in 2020 yielded compound **15**. The compound retained slight HDAC1 preference but dropped in overall potency, exhibiting an IC_50_ of 1.07 µM. Seemingly, the dropped in vitro potency had a negligible effect on cell assays, exhibiting GI_50_ values of 12 nM and 22 nM against KB-7D and KB-Vin cell lines. Such unexpected results are sometimes observed for compounds with multiple mechanisms of action. In 2017, Xie et al. [87] developed amino benzamide HDACi **16**, which inhibited HDAC2 with an IC_50_ of 570 nM and was potent against diverse cancer cell lines with activities ranging from 3.84–5.37 µM. Unfortunately, further in vivo evaluation showed poor pharmacokinetic potential with a short half-life and fast metabolism. Slight modifications by Yun et al. [88] in 2019 yielded **17a**, **b** with increased potency against HDAC1 (16 nM and 71 nM) and improved pharmacokinetic properties. Inhibition of solid cancer cell lines A549, A375, SMMC7721, HCT116, and Hela was in the range of 1.64–3.01 µM and 1.74–3.0 µM for compounds **17a** and **17b**, respectively. Another study by Cheng et al. [89] in 2019 yielded several inhibitors such as **18** with a thioquinazolinone cap. Four out of 40 tested compounds seemed to have promising inhibitor activities in the range of 10–160 nM against HDAC1 and HDAC2, and some showed up to 4000-fold selectivity over HDAC6. Experiments with A375, Hela, A549, HCT116, and SMMC7721 cell lines showed potent antitumor efficacy with low toxicity against NIH 3T3 cell lines (2.5–5 µM) and promoted cell apoptosis more potently compared to reference compounds such as entinostat. In 2017, Abdizadeh et al. [90] synthesized coumarine cap-based amino benzamides and evaluated them against several cancer cell lines. Among them, four compounds (**19a–d**) were most promising, with **19d** being most potent for HDAC1 (470 nM). Compound **19d** induced cytotoxicity in several cancer cell lines with IC_50_ values of 10.41 µM (MCF7), 4.18 µM (A549), 22.72 µM (PC3), and 15.77 µM (HL-60) and had no effect on HUVEC viability with an IC_50_ > 100 µM. Various potential HDACis were tested by Wang et al. [91] by connecting a protected lysine residue with different ZBGs. Compound **20** containing an amino benzamide moiety was one of the most potent. In 2015, Li et al. [92] searched for stable and pharmacodynamically favorable HDACis aiming at the installation of therapeutically beneficial cap groups and designed imidazopyridine and -pyridazine moieties. These were evaluated against HDAC1 and several cancer cell lines. Compound **21** was most potent, inhibiting HDAC1 with 118 nM and exhibiting IC_50_ values of 1.258 µM (HCT-116), 2.871 µM (MCF-7), and 723 nM (A549), being twofold more potent than reference compound entinostat and exhibiting fivefold enhanced cytotoxicity against the A549 cell line. Sufficient oral bioavailability suggested further development of the compound. In summary, amino benzamides are one of the best studied warheads, with the clinically approved representative chidamide. Classic benzamides typically show long residence times and exhibit an intrinsic class I selectivity, which is further adjustable toward HDAC1 and HDAC2 within class I isozymes. Altogether the amino benzamide ZBG is a valuable scaffold for several clinical and research-related applications. 

## 4. Non-Classic Benzamides

**Figure 7:**
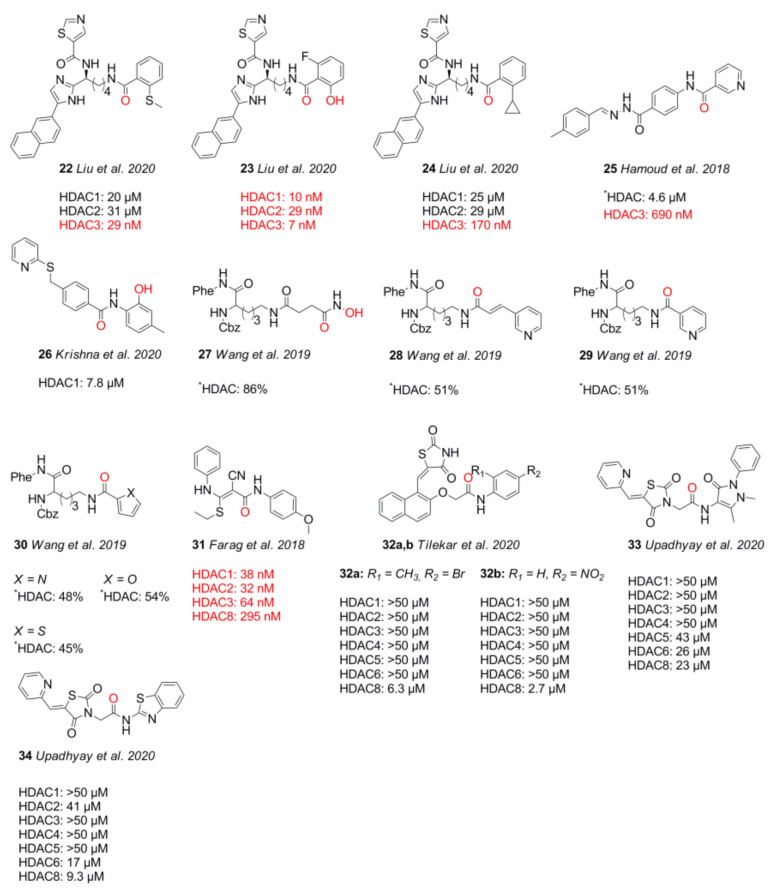
Non-classic benzamide compounds prepared in the listed studies. Chelating atoms, suggested by solved crystal structures or docking and IC_50_ values below 1 µM are highlighted in red. * Inhibitory activity is given as the percentage inhibition of HeLa nuclear extract as an HDAC source in the presence of **25** (10 µM) and **27**, **28**, **29**, and **30** (100 µM).

**Figure 8:**
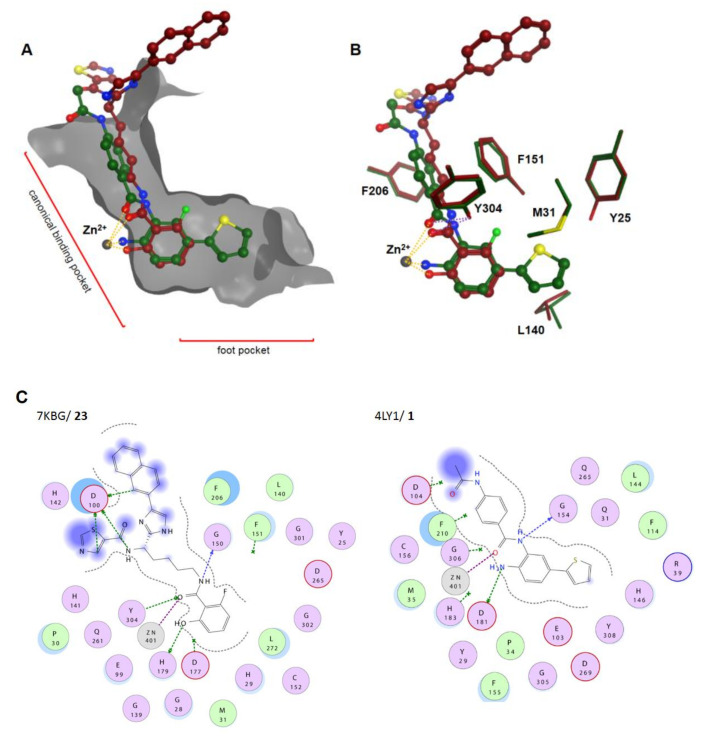
(**A**) Overlay of HDAC2 crystal structures (PDB ID: 7KBG/red and 4LY1/green) of classic and non-classic benzamide derivatives **1** and **23** with a clipped gray surface and the zinc ion as a gray sphere. (**B**) Visualization of residues in proximity to the foot pocket. Notably, classic and non-classic benzamides exhibit similar binding modes with no major distortion of residues within the foot pocket. Contrarily, methylated derivatives seem to adopt a distorted conformation by inducing rotameric changes (not shown). (**C**) The 2D ligand interactions of in (**A**) overlaid crystal structures with PDB IDs 7KBG and 4LY1, respectively.

**Figure 9:**
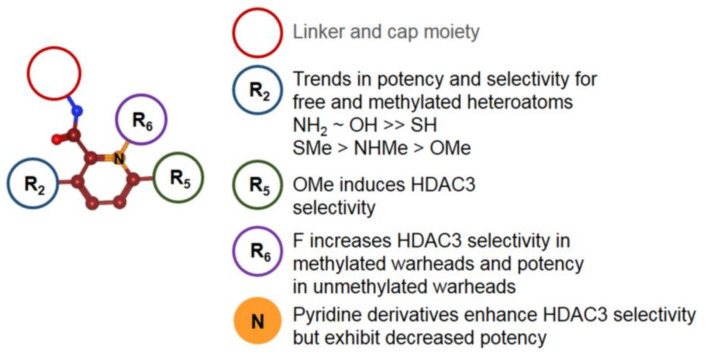
Schematic SAR of amino benzamide warheads studied by Liu et al. [76] with indicated spheres as placeholders for substitution.

Compounds with similar ZBGs to classic benzamides include non-classic benzamide and aryl amide derivatives such as **22–34**, which differ in several aspects compared to classic benzamides (Figure 7). In contrast to classic benzamides, the chelation proceeds in an equipotent manner through the carbonyl moiety if no other chelating atom is present, as illustrated by compounds **22**, **24**, and **25**. Unlike classic benzamides, warheads of compounds **26–34** comprising a non-classic benzamide ZBG or an aryl/heteroaryl moiety are more diverse, showing IC_50_ values with a wide nM to µM range. Similarly to classic amino benzamides, compounds with these warheads are predominantly class I-selective with preference for HDAC3 and HDAC8, complementing classic benzamide HDACi. Special emphasis should be placed on the series of Liu et al. [76] with inhibition in the nM range and long residence times of up to 69.4 h for compounds such as **23**. The authors suggested that the much shorter residence time observed for compound **22** is due to a lack of polar interactions, leading to a strongly decreased residence time of 38 min. In contrast to the slow on-rate of classic amino benzamides caused by the intramolecular H-bond between the carbonyl oxygen and the aromatic *ortho* amino group, the presented warheads herein showed a fourfold faster on-rate of 1 × 10^5^ M⋅min^−1^. Unfortunately, the compounds of this series suffered from high clearance and off-target effects. Based on crystal structure data from Liu et al. [76], an insight into properties was provided. Noteworthy are the shorter interaction distances between the zinc ion and the chelating groups of **22** and **23**, ranging from 2.0 Å to 2.2 Å for the (OH) and (CO) moieties compared to classic benzamides with distances of 2.1 Å (NH_2_) to 2.46 Å (CO). This can be reasoned as a consequence of bond and angle properties between the chelating atoms, with shorter distances of four bonds for non-classic and longer distances of five bonds for classic benzamides (Figure 8A–C). A short SAR analysis based on studies by Liu et al. [76] is illustrated in Figure 9. Methylation of the heteroatom at position R_2_ induced strong HDAC3 selectivity in the order SMe > NHMe > OMe. Interestingly, potency follows a reversed order for non-methylated heteroatoms, NH_2_ ≈ OH >> SH. Substitution at R_5_ was sparely evaluated but stirred toward a fivefold increase in HDAC3 selectivity with a fourfold loss in potency. Likewise, substitution at R_6_ could not be fully evaluated, pointing to an increase in selectivity toward HDAC3 in combination with methylated heteroatoms and a gain in potency when present in the non-methylated heteroatom derivatives [76]. Pyridine derivatives show a small gain of selectivity for HDAC3 accompanied by a penalty in potency. Altogether derivatization at position R_5_ and R_6_ goes along with minor selectivity wins and major potency losses, while methylation of heteroatoms at position R_2_ confers remarkable selectivity toward HDAC3.

In an effort to identify novel ZBGs, Liu et al. [76] tested several benzamide derivatives and discovered highly potent and selective 2-substituted benzamides **22** and **23**. Compound **22** was able to inhibit HDAC3 with an IC_50_ of 29 nM and exhibited over 300-fold selectivity over all other isoforms. Strikingly compound **23** was equipotent but lacked selectivity. This finding could be rationalized by X-ray crystal structures of compounds **22** and **23** bound to wild-type and insertion mutant HDAC2. The insertion mutant Y205, designed to better reflect the active site of HDAC3, exhibited a rotameric Y305 “in” to “out” change. This induced change led to pocket expansion and ensured a better fitting of the sterically more demanding 2-methylthio benzamide ZBGs, which coordinates the zinc with the amide carbonyl in a monodentate fashion. Altogether, the authors suggested that the observed selectivity toward HDAC3 originates from the energetic cost for structural rearrangements in HDAC1 and HDAC2, which are required to accommodate the sterically more demanding compound. This conception is supported by potent and selective binding of **24** to HDAC3 but is counterintuitive regarding the much faster on-kinetic and a 110-fold lower residence time of 38 min for compound **22** compared to **23**, considering the need for conformational changes. Intravenous administration of 0.05 mg⋅kg^−1^ did not yield detectable levels of compound due to clearance. Off-target studies of **22** showed adverse effects by inhibiting the potassium ion channel hERG with an IC_50_ of 16 nM. This inhibitory potential against hERG could be minimized to 1.33 µM by applying derivatization strategies from similar studies [93]. Hamoud et al. [94] used the nicotinamide moiety as an alternative ZBG and varied the cap group. They could show that compound **25** was the most potent in the series, showing an inhibitory activity of 4.64 µM against Hela nuclear extract and 690 nM toward HDAC3. Further testing in cancer cell lines showed IC_50_ values of 4.66 µM (B16F10), 9.45 µM (MCF-7), and 14.81 µM (A549), as well as no significant toxicity against normal human embryonic kidney HEK-293 (169.5 µM) cells. Further in silico studies indicated good drug-like properties and a monodentate binding of **25**. Krishna et al. [95] identified several compounds using in silico screening and evaluated the most promising in the human breast adenocarcinoma MDA-MB-231 cell line. Inhibitory activity of **26** against HDAC1 was determined to be 7.81 µM. Unfortunately, compound **26** had only weak inhibitory activity against the tested cancer cell line. The aforementioned screening by Wang et al. [91] (Figure 5, compound **20**) using arylamide derivatives as ZBGs attached to a lysine derivative yielded hydroxamate **27** as a positive control, as well as pyridine derivatives **28** and **29** and five-membered heterocycles **30a–c**. In 2018, Farag et al. [96] synthesized multiple compounds, among which the benzamide analog **31** exhibited class I selectivity, as well as HDAC2 preference, with an IC_50_ of 32 nM. Further screening against 60 cancer cell lines (NCI-60) showed activity against leukemia and melanoma cell lines with GI_50_ values of 2.87 µM (HL-60) and 370 nM (MDA-MB-435). Tilekar et al. [97] searched for HDAC8-selective non-hydroxamate inhibitors and designed a series of 2,4-thiazolidinedione-containing compounds based on earlier studies. The series showed IC_50_ values in the 2.7–12 µM range and antiproliferative activities in the range of 420 nM–2.05 µM and 13.94–39.79 µM for K562 (chronic myeloid leukemia) and CEM (lymphoblastic leukemia) cancer cell lines, respectively. The most promising compounds **32a**, **b** showed IC_50_ values of 6.3 µM and 2.7 µM (HDAC8) and >50 µM for other HDAC isoforms (HDAC1-6). Thermal shift assay (TSA) determined a significant stabilization of the protein upon binding of the inhibitor (∆T_m_ = 7 °C). Evaluation of **32a** in cancer cell lines K562 and CEM showed apoptosis and cell-cycle arrest in the G2/M phase with IC_50_ values of 2.05 µM and 15.71 µM, as well as no inhibition of normal blood cells (WBC—152 µM) [97]. In contrast to the assumed chelation by the thiazolidinedione moiety as in previous studies [98], docking results suggested chelation via the amide carbonyl. Further studies by Upadhyay et al. [99] were also aimed at the design of novel ZBGs with the thiazolidinedione motif and yielded compounds **33** and **34** as the most potent and selective inhibitors of the series for HDAC8. Compounds **33** and **34** showed an inhibitory activity of 23 µM and 9.3 µM against HDAC8, as well as 26 µM and 17 µM against HDAC6, but no potency >40 µM for other isoenzymes. Evaluation of **34** in three cancer cell lines (CEM, K562, and KCL22) showed CC_50_ values of 79.9 µM, 85.4 µM, and 43.2 µM. Two different viability assays suggested only low cytotoxicity against noncancerous WBC cells. Computational predictions suggested drug likeness and good passive oral absorption but bad metabolic stability. Additional evaluation of the series against glucose transporters showed that only compound **34** exhibited inhibition of GLUT1 with an IC_50_ value of 28.2 µM, not affecting GLUT4 or GLUT5. Taken together, non-classic benzamide warheads include a greater variety of ZBGs and exhibit mainly class I selectivity. Outstanding is the complementary HDAC3 selectivity of **22** compared to classic benzamides, indicating an exploitable mechanism.

## 5. Amide Warheads

**Figure 10:**
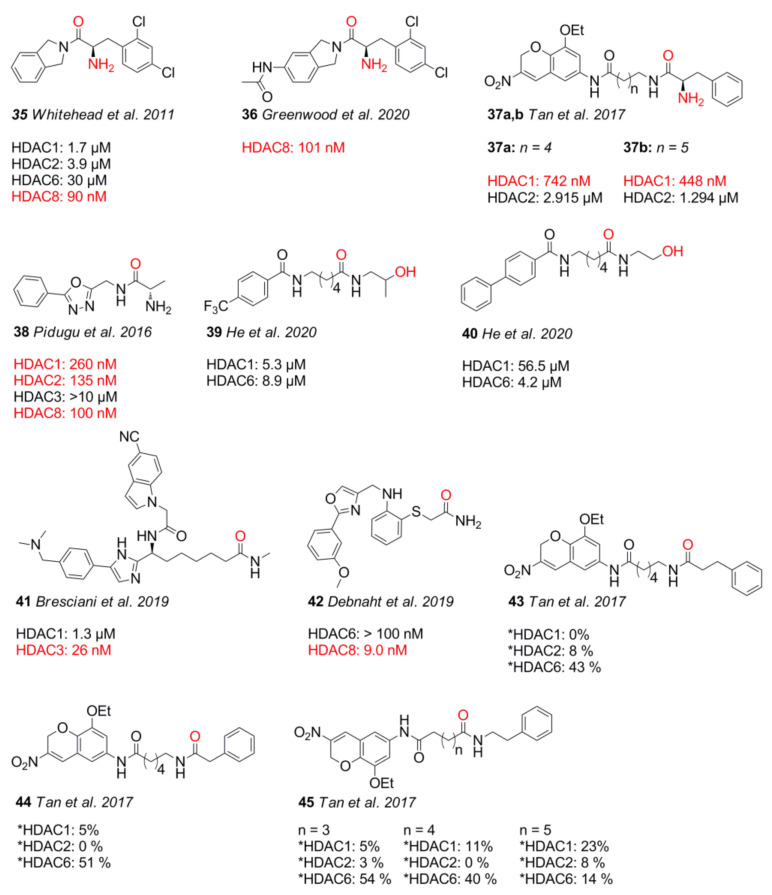
Amide-containing HDACis with proposed chelating atoms and low IC_50_ values under 1 µM are highlighted in red. * Inhibitory potency is given as the percentage inhibition of HDACs in the presence of 10 µM compound.

Further related scaffolds include various amides with diverse substitution patterns, which are mostly class I-selective. One of the most potent motifs is the α-amino amide, present in compounds **35**, **36**, **37**, and **38** (Figure 10). In addition to α-amino amides, compounds **39** and **40** chelate via a hydroxy amide motif and show interesting selectivity properties [100]. 

Unfortunately, no pharmacokinetic or stability data could be found for any compounds but **41**, which was stable in human and mouse plasma, as well as hepatocytes (t_1/2_ = 4 h) [101]. 

**Figure 11:**
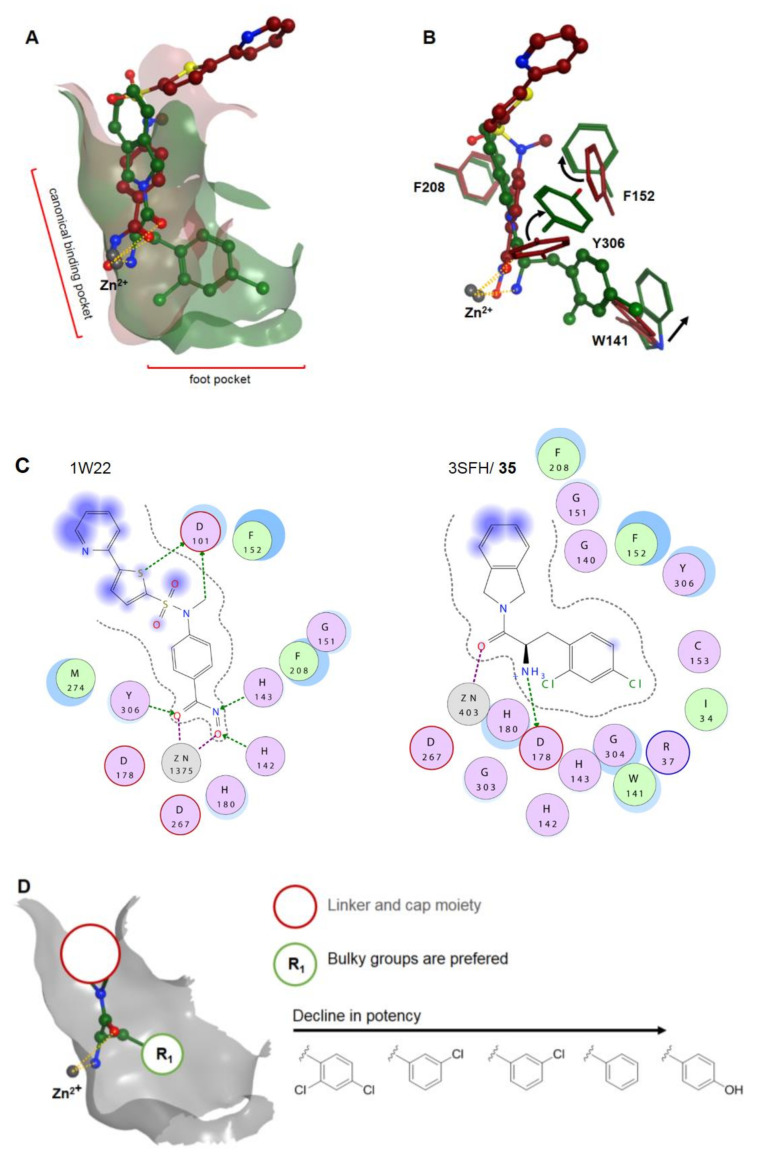
(**A**) Structural overlay of HDAC8 complexed with the α-amino amide **35** (PDB ID: 3FSH/green) and a hydroxamic acid (PDB ID: 1W22/red). Shown is the clipped binding pocket and the foot pocket with the zinc ion as a gray sphere and metal contacts as dotted orange lines. (**B**) Conformational changes leading to the enlargement of the foot pocket. W141 rotates away, enlarging the lower half, and Y306 and F152 rotate in a codependent manner enlarging the upper half of the foot pocket. (**C**) The 2D ligand interactions of in (**A**) overlaid crystal structures of a hydroxamic acid and **35** with PDB IDs 1W22 and 3SFH, respectively. (**D**) Binding mode of α-amino amides and indicated potency trends for bulky substituents. Metal bonds are shown as dotted orange lines.

As expected, α-amino amides tend to chelate the zinc ion in a bidentate fashion with IC_50_ values in the nM range and interaction distances of 2.3 Å and 3.0 Å for the amine and the carbonyl moieties, respectively [102]. Monodentate species such as **41** and **42** are also potent inhibitors with activity in the nM range [101,103]. Compounds **43–45** exhibited poor potency against the tested HDAC1, HDAC2, and HDAC6 isoforms, but had better antiproliferative activities than the amino benzamide and α-amino amide ZBGs, suggesting additional mechanisms of action [80]. The canonical binding pocket of HDAC8 is highly moldable, allowing for the accommodation of small structures such as SAHA and sterically demanding moieties such as **35** (Figure 11A,B). The residues Y306, F152, W141, and I34 in HDAC8 (PDB IDs: 1W22 and 3FSH) limit the pocket size much in the same way as residues L140 and M31 do in HDAC2, but they undergo a much greater displacement. A visualization of HDAC8 and a brief SAR of the α-amino amide class is shown in Figure 11. Efforts to improve **35** were explored only to find that the linker cannot easily be replaced or modified to yield more potent derivatives [102,104]. The same was found to be true for substitutions at R_1_, leading to less potent compounds compared to **35**. Studies by Tan et al. [80] underlined the strong complexation ability of the amine moiety through a comparison of **37a** and **43**. Compound **38** is special in regard to its stereochemistry compared to other α-amino amides. 

In 2020, Greenwood et al. [104] conducted an SAR study on an the α-amino amide HDACi **35**, formerly reported by Whitehead et al. [102] in 2011. To explore the role and relative importance of substitutions at R_1_, also called leader groups or linker groups, analogs of **35** were prepared and screened against HDAC8 with the most potent compound **36**, having a twofold higher potency of 101 nM compared to **35**, which showed an activity of 210 nM in the given screen. Analysis of several leader groups confirmed the 2,4-dichlorphenyl moiety to be essential with a binding energy gain of 4.3 kJ⋅mol^−1^ compared to a plain phenyl moiety. Incorporation of amides or amines into the isoindoline group induced hydrogen bonding to D101 near the channel exit and retained or even improved potency [104]. In addition to the aforementioned amino benzyl amides, Tan et al. also synthesized 3-nitro-2*H*-chromene-capped α-amino amides **37a** and **37b** [80]. Compound **37a** and **37b** had an inhibitory activity of 742 nM and 448 nM toward HDAC1, as well as 2.915 µM and 1.294 µM toward HDAC2, respectively. In vitro data of compound **37a** showed better or comparable antiproliferative activity to reference compounds SAHA and entinostat with GI_50_ values of 3.56 µM (K562), 5.95 µM (A549), 8.72 µM (MCF-7), 11.17 µM (PC-3), and >20 µM (Hela). Other amide derivatives **43–45** achieved even slightly better antiproliferative results in tested cell lines than amino benzylamides and α-amino amides, but they showed only moderate potency toward HDAC6 or other isoforms. In the search of improved HDAC8 inhibitors, Pidugu et al. [105] designed and evaluated 2,5-disubstituted 1,3,4-oxadiazoles based on earlier studies from Valente et al. [106] and Rajak et al. [107]. In silico studies identified suitable compounds with an α-amino amide ZBG, which were synthesized and tested. Compound **38** showed an inhibitory activity of 260 nM (HDAC1), 135 nM (HDAC2), and 10 µM (HDAC3), as well as a preference for HDAC8 (100 nM), and it was most potent against MDA-MB-231 breast cancer cells with an IC_50_ of 230 nM. Docking results of **38** indicated only monodentate chelation differing from other HDAC8 selective α-amino amides such as **35** [102] and **36** [104] and eventually suggesting a different chelation mode depending on chirality [105]. Class IIb selective HDAC6 inhibitors with µM range potencies were synthesized by He et al. [100] and tested against HDAC isoforms and cancer cell lines. Compounds **39** and **40** showed a potency of 5.3 µM and 56.5 µM toward HDAC1, as well as 8.9 µM and 4.2 µM toward HDAC6, respectively. Both compounds contain a 2-hydroxy alkyl amide ZBG. Noteworthily, compound **39** shows lesser selectivity compared to **40** albeit more sterically demanding. This could be rationalized by comparing docking poses of **39** and **40** in HDAC1 and HDAC6. Docking into HDAC6 suggested a bidentate binding mode for both compounds [100]. Docking in HDAC1 suggested a monodentate binding mode of **40**. The authors argue that the cap group is most important for selectivity by displacing the linker moiety, thereby negatively affecting the chelation mode of **40** toward HDAC1. Cell line experiments with **40** showed antiproliferative activity in the range between 13.6 and 40.0 µM toward A549, HepG2, Hela, and MCF-7 tumor cell lines, as well as 95.9 µM toward normal cell line human lung fibroblast WI-38. On the basis of previous research [108], Bresciani et al. [101] reported a set of HDAC3-selective inhibitors that performed better than the commonly used tool compound RGFP996. Compound **41** had 50-fold selectivity over HDAC3 and exhibited an inhibitory activity of 26 nM for HDAC3 and 1.30 µM for HDAC1. It had good stability in vitro, exhibiting a half-life of greater than 4 h in plasma, as well as in hepatocytes from mouse and human. Debnaht et al. [103] generated a 3D QSAR model based on 32 known and selective HDAC8 inhibitors and screened the Phase database to identify five amide analogs for in vitro testing. Compound **42** exhibited an acetamide ZBG and was a potent HDAC8 inhibitor with an IC_50_ of 9.0 nM. According to docking data, **42** is suggested to chelate the zinc ion predominantly through the amide carbonyl group. In summary, α-amino amide warheads are a diverse and complementary part of a bigger family (classic and non-classic benzamides) in terms of structure and HDAC8 potency and selectivity. Unfortunately, the latest studies [104] indicate an already optimized structural space, which is not easy to further improve. A lack of pharmacokinetic data additionally impedes full assessment. Other amide-containing warheads such as the series of He et al. [100] display interesting properties, which can be further exploited to achieve potent and selective HDAC6 inhibitors. 

## 6. Hydrazide Warheads

**Figure 12:**
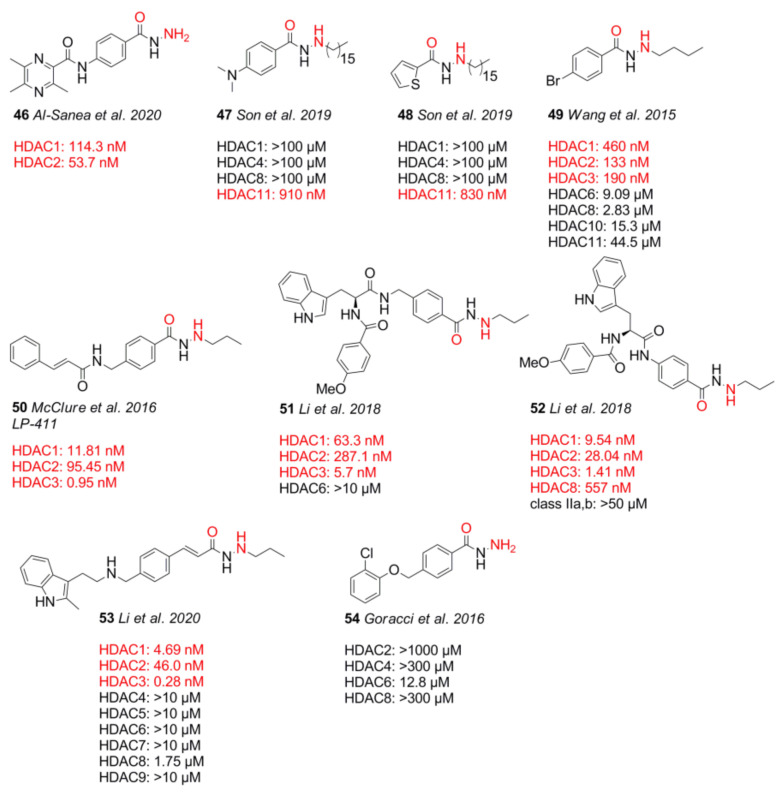
Hydrazide warhead-containing HDACis. Proposed chelating atoms and IC_50_ values below 1 µM are highlighted in red.

Hydrazides belong to one of the latest discovered warheads and exhibit HDAC class I (HDAC1–3) preference (Figure 12). Further properties include high potency in the low nM range, beneficial fast-on/slow-off kinetics with residence times up to 96 h, and metabolic stability with half-times of over 15 h [109]. 

**Figure 13:**
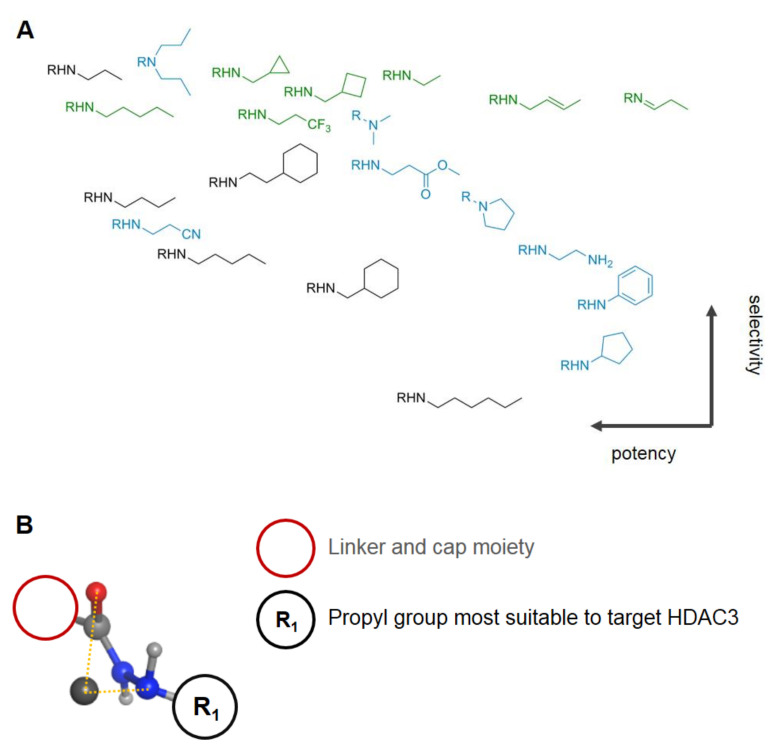
(**A**) Schematic potency trends are given on the basis of studies by McClure et al. [114] (green), Wang et al. [109] (black), and Li et al. [110,111] (blue). (**B**) Li et al. [111] through docking suggested the binding mode of hydrazides with the zinc ion as a gray sphere and metal bonds indicated as dotted orange lines. Substitutions are indicated by spheres.

In addition, this warhead is negative in the Ames test, a bacterial mutagenicity test, is stable toward glucuronidation, and exhibits better oral bioavailability with over twofold higher area under curve (AUC) values compared to hydroxamates [110]. Further advantages are the well-known toxicity and safety profile of several FDA-approved drugs such as isoniazid and phenelzine, which incorporate structurally related hydrazine and hydrazide motifs. The hepatotoxicity of FDA-approved isoniazid has been observed in up to 4% cases with up to 0.1% lethal cases according to [33]. Docking results by Li et al. [110,111] suggest a bidentate chelation of alkyl hydrazides, with reasonable distances between the ZBG and the zinc ion. This is also considered true for bare hydrazide moieties [112,113]. The predominant leader group of hydrazide motifs is a propyl moiety, which is shown to be most potent and selective toward HDAC3 (Figure 13). Sterically more demanding groups such as cyclopropyl methyl or cyclobutyl methyl mimicking the propyl scaffold have comparable selectivity but a decreased potency. On the basis of studies from McClure et al. [114], Wang et al. [109], and Li et al. [110,111], it is evident that constrained leader groups incorporating cyclic motifs or unsaturated bonds are inferior to alkyl chains in selectivity and potency. A contrary example to the trend of inferior performance with steric demand and rigidity is a double-substituted propyl moiety, which shows 16-fold selectivity over HDAC1 and potency in the nM range [110]. 

Al-Sanea et al. [113] combined hydrazides with a ligustrazin cap to yield **46** (Figure 13), the most potent compound of this series. Compound **46** showed inhibitory activity of 114.3 nM and 53.7 nM toward HDAC1 and HDAC2 and had a potency of 15.10 µM and 1.60 µM toward HT-29 and SH-SY5Y cells, respectively. Son et al. [115] developed a series of HDAC11-specific inhibitors based on known HDAC1 inhibitors and, in addition, incorporated long-chain moieties which are preferred by HDAC11. Inhibitory potential was determined via an enzyme activity assay with *n*-tetradecanoyl peptide substrates, which could be separated and analyzed via HPLC upon enzymatic cleavage. Compounds **47** and **48** were found to inhibit HDAC11 in the upper nM range with IC_50_ values of 910 nM and 830 nM, respectively, comparable to the positive control FT895 with an IC_50_ value of 740 nM. Wang et al. [109] reported an inhibitor of cancer cell proliferation by induction of cell death, as well as suppression of cell-cycle progression and DNA repair. Cell experiments showed that **49** had fast-on and slow-off kinetics inducing histone acetylation within 6 h and maintaining it for 96 h, as well as showing a half-life of 15.8 h in a cell culture medium containing 10% fetal bovine serum. Lead compound **49** showed IC_50_ values of 460 nM (HDAC1), 133 nM (HDAC2), 190 nM (HDAC3), 2.83 µM (HDAC8), 9.09 µM (HDAC6), 15.3 µM (HDAC10), and 44.5 µM (HDAC11), as well as >100 µM (HDAC4, 5, 7, 9). Continuing work by McClure et al. [114] aimed at developing a non-hydroxamate inhibitor to combat leukemia with emphasis on HDAC3 selectivity as it controls hematopoiesis. Compound **50** was most promising and inhibited HDAC isoforms with IC_50_ values of 11.81 nM (HDAC1), 95.45 nM (HDAC2), and 0.95 nM (HDAC3). Selected hydrazide-based inhibitors were exposed to glucuronidating conditions ex vivo and showed no glucuronidation. Lineweaver–Burk plots indicated mixed inhibition, pointing toward an allosteric binding site near the NCOR2 binding interface as suggested by docking. Evaluation in cancer cell lines yielded EC_50_ values of 36.37 nM (MV4-11), 76.64 nM (Molm14), 151.7 nM (RS4-11), 2.16 µM (K562), >10 µM (HL-60), and >10 µM (RPMI-8226) [114]. Later on, Li et al. [111] introduced a hydrazide warhead into a modified lead compound creating a new series [111]. Compound **51** was the most selective with an IC_50_ value of 63.28 nM (HDAC1), 287.1 nM (HDAC2), and 5.63 nM (HDAC3), showing an 11- and 51-fold higher selectivity against HDAC1 and HDAC2, respectively, and exhibiting a slow and tight binding inhibition mechanism. Lineweaver–Burk analysis of in vitro studies indicated mixed inhibition by compound **51**, which suggested an allosteric binding pocket in heterodimer HDACs based on previous studies and docking analysis [111,114]. Compound **52** was the most potent with an inhibitory activity of 9.54 nM (HDAC1), 28.04 nM (HDAC2), and 1.41 nM (HDAC3) and showed an EC_50_ value of 34.7 nM against MV4-11 cells. Noteworthily, there are different antiproliferative mechanisms of **52** toward p53-wt and p53-null cells, showing p53-dependent apoptotic pathway induction and a cytostatic effect by G2/M arrest. Further studies by Li et al. [110] led to the panobinostat-based compound **53**. This lead compound showed low nanomolar IC_50_ values of 4.69 nM (HDAC1), 46.0 nM (HDAC2), 0.28 nM (HDAC3), 1.75 µM (HDAC8), and >10 µM (HDAC4–9) and exhibited cell-dependent toxicity profiles. Compound **53** showed overall better pharmacokinetic properties compared to the control panobinostat which included a fivefold higher bioavailability and half-lives of 15.2 h (intravenous) and 7.45 h (oral). Additionally, **53** did not exhibit mutagenic toxicity in a mini-Ames test. In 2016, Goracci et al. [112] identified an HDAC6-selective hydrazide inhibitor. The authors attributed the origin of HDAC6 selectivity to the linker and cap group [112]. The most potent compound **54** inhibited HDAC6 with only 12.8 µM and increased levels of acetylated alpha-tubulin with no effects on histone H4 acetylation. Furthermore, **54** showed low cytotoxicity toward Hela and HEK 293 cells but had a good potential to cross the blood–brain barrier; therefore, it was suggested as a promising starting point. To sum up, hydrazide warheads are some of the most recently discovered ZBGs and have very promising pharmacokinetic properties, particularly fast-on/slow off-rates, long residence times, good stability, and no mutagenic potential; moreover, structurally related and FDA-approved drugs make this class very appealing and promising. 

## 7. Ketone Warheads

**Figure 14:**
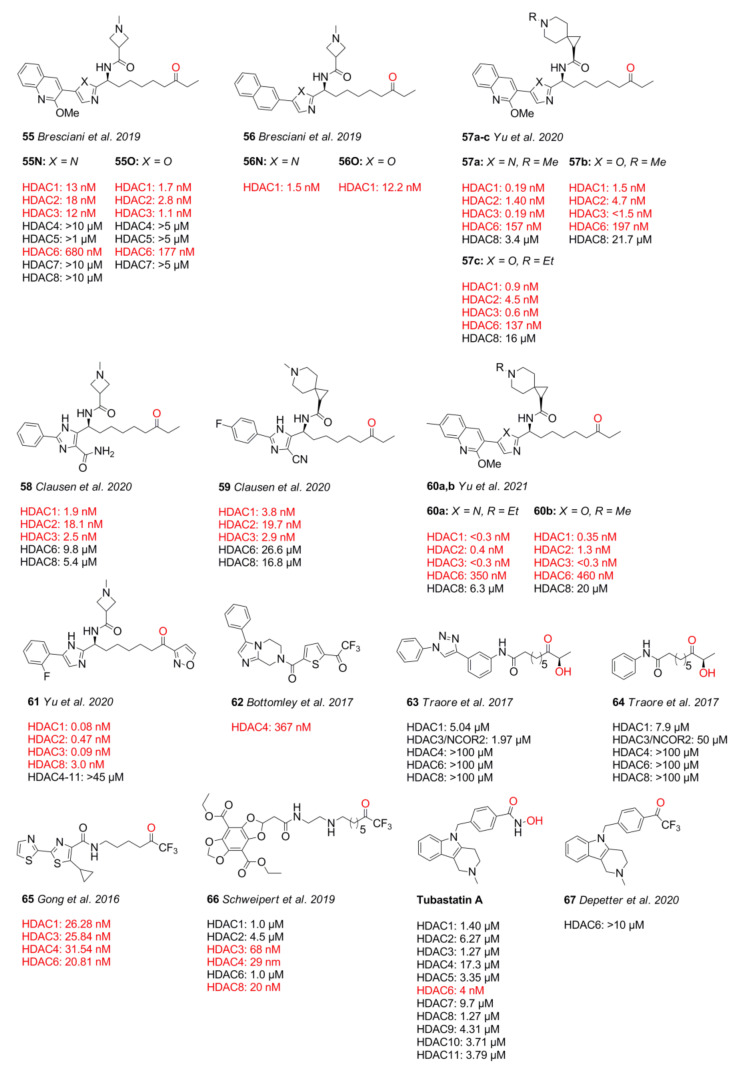
HDACi with a ketone warhead. Chelating atoms and inhibitory activity below 1 µM are highlighted in red.

**Figure 15:**
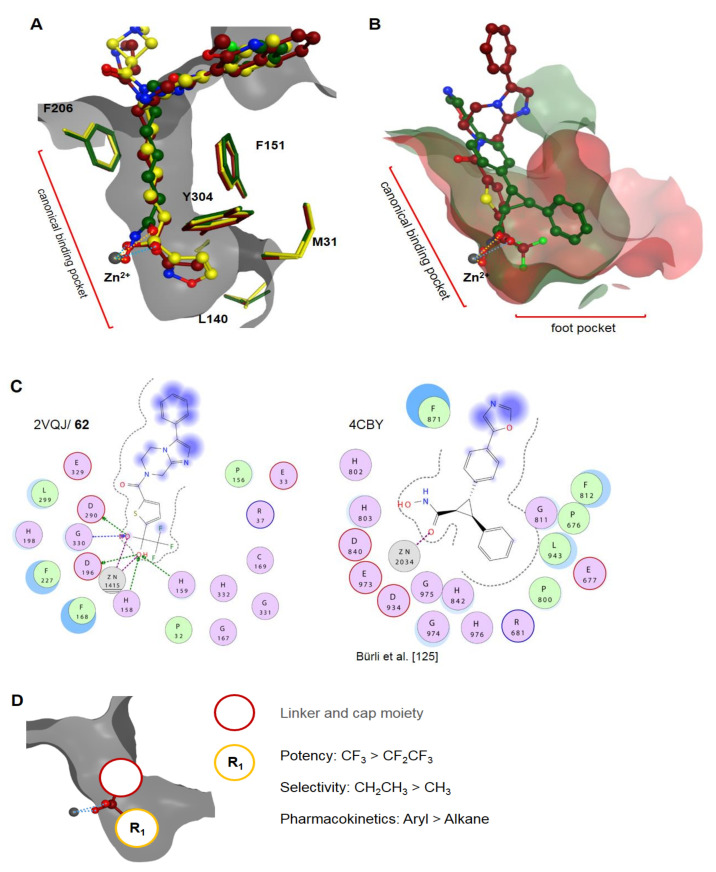
(**A**) Structural overlay of HDAC2 complexed with ketones **55** and **61** (PDB ID: 6XDM/yellow and 6WBW/red) and a hydroxamate (PDB ID: 4LXZ/green). Shown is the clipped binding pocket with the zinc ion as a gray sphere and metal contacts in yellow and blue for the hydroxamate and the diol moiety, respectively. (**B**) Structural overlay of HDAC4 complexed with a hydroxamate prepared by Bürli et al. [125] in a closed conformation (4CBY/green) and **62** by Bottomley et al. [124] in an open conformation (2VQJ/red), indicating large induced conformational changes of the foot pocket, which might be exploitable for further drug and warhead design. (**C**) Ligand interactions of the hydroxamic acid by Bürli et al. [125] and **62** within the canonical binding site of HDAC4. (**D**) Binding mode of ketones with a short SAR summary for the warhead.

Ketone-based warheads exhibit a strong inhibitory potential by chelating the zinc ion through a germinal diol moiety, which is in equilibrium with the ketone and is affected by substitution [116]. Ketones equipped with an electron-withdrawing substituent such as fluorine are increased in electrophilic nature, thus being readily hydrated, forming the active, transition state mimicking diol at physiological pH [116]. Ketone-based warheads tend to exhibit lower selectivity compared to other ZBGs, inhibiting class I and class IIa, b in the nM range with selectivities ranging from 1 to >10,000, depending on the cap group and the nature of the ketone (Figure 14). Ketone warheads exhibit fast on- and off-kinetics with mean residence times of 5 min to 3 h, ranging up to 40 h, depending highly on secondary interactions and steric demand of the cap group and leader group [93,117]. Unfortunately, ketone warheads exhibit bad pharmacokinetic properties in general. Most are unstable in blood plasma with half-lives of under 15 min and are rapidly metabolized in cells by carbonyl reductases to the corresponding alcohol [118,119,120]. According to Frey et al. [119], metabolization is especially dominant for sterically undemanding straight alkyl chains. Several steric and electronic transformations seem not to improve the metabolic instability [119]. Current compounds from Kozlowski’s group show a wide range of plasma half-lives ranging from 2–43 h and moderate clearance of 6–60 mL⋅min^−1^⋅kg^−1^ [93,121]. Geminal diols show comparable metal bond distances of 2.02 Å and 2.42 Å (PDB IDs: 2GH6, 2VQJ, 2VQO, 6XDM, and 6WBW) to hydroxamates, as well as 2.0 Å and 2.28 Å (PDB ID: 4LZX), and to free acetate with 2.18 Å and 2.3 Å (PDB ID: 7LTL). Similarly, the small steric demand, especially of trifluoromethyl ketones and hydroxamates, leads to isoform selectivity issues [122,123]. Sterically somewhat more demanding ethyl- and arylketone warheads such as in **55–60** and **61** and derivatives thereof (not shown) show only a preference for HDAC1–3 but not a profound selectivity over HDAC6 and HDAC8, requiring optimization of the cap group to achieve isoform selectivity (Figure 15A). Notably, trifluoromethyl ketone **62** from Bottomley et al. [124] was selective toward HDAC4 (class IIa) and could be crystalized in an open conformation of the protein (Figure 15B,C), which is, to this date, the only reported open structure of this protein class. Structurally divergent compounds such as α-hydroxy ketones **63** and **64** are not as potent or inherently selective, inhibiting in the µM range, and they are structurally reminiscent of α-amino amides, which are not included in the schematic SAR illustration presented in Figure 15D.

Electrophilic ketones lose potency with increasing steric demand, showing the highest inhibitory activity for trifluoromethyl compared to pentafluoroethyl moieties [126]. This potency loss does not apply to alkanes and favors the use of ethylketones with better selectivity over HDAC6 and slightly higher potency in vitro [108]. Sterically more demanding five-membered azole moieties such as **61** exhibit a tighter fit and add to pharmacokinetic properties. Further substitutions on the aryl moiety seem to induce HDAC3 selectivity, accompanied by a loss in potency [117]. 

Together with methyl amides (Figure 10, compound **41**), Bresciani et al. [101] also synthesized ketone analogs. The authors suggested minor cap group-dependent potencies (Figure 14, **55** and **56**). While using 2-methoxyquinoline as a cap group, the imidazole core moiety in **55** had an IC_50_ value of 13 nM compared to oxazole (1.7 nM); exchanging the 2-methoxyquinoline cap with a naphtyl cap reversed the trend and yielded imidazole compounds **56** with IC_50_ values of 1.5 nM compared to oxazole (12.2 nM). Compound **55O** showed nM inhibition toward HDAC1 (1.7 nM), HDAC2 (2.8 nM), and HDAC3 (1.1 nM), as well as profound selectivity over HDAC6 (177 nM) and inactivity toward HDAC4–7 at 5 µM. Genotoxicity assessments of **55O** highlighted the Ames-negative profile and metabolic inactivity toward four cytochrome p450 family members with values above 20 µM. Pharmacokinetic analysis showed an improved profile of oxazole compared to imidazole moieties with medium–high blood–brain barrier permeability in porcine brain endothelial cells and high to modest in vivo bioavailability, as well as a plasma half-life of 3.3–3.7 h and moderate clearance (20 mL⋅min^−1^⋅kg^−1^) [101]. Similar scaffolds were used by Yu et al. [121] and Clausen et al. [127] for HIV treatment. In the “shock and kill” strategy, HDACs acts as the “shock”, potentially reactivating the latent HIV reservoir and, thus, inducing the HIV gene transcription in resting cells, making them susceptible to “kill” strategies. Yu et al. [121] reported compounds **57a–c** with nM potency, high selectivity, and reasonable pharmacokinetic properties with half-lives of 3.0–8.6 h, oral bioavailability of 6–69%, and a C_max_ of 0.26–1.43 µM. Compound **57a** was most potent with IC_50_ values of 0.19 nM (HDAC1), 1.4 nM (HDAC2), and 0.19 nM (HDAC3). As Bresciani et al. [101] noticed and Yu et al. [121] calculated, oxazole derivatives **57b** and **57c** exhibited twofold better permeability, explaining the improved pharmacokinetic properties. In 2020, Clausen et al. [127] identified **58** and **59** as lead compounds and determined their pharmacokinetic properties in rats. Compounds **58** and **59** showed very good inhibitory activity of 1.9 nM and 3.8 nM (HDAC1), 18.1 nM and 19.7 nM (HDAC2), and 2.5 nM and 2.9 nM (HDAC3), respectively. Due to their high polarity, these were most suited for intravenous dosing, exhibiting good half-life values of 6.4–4 h and low clearance. Further studies in the field of HIV latency reactivation by Yu et al. [93] were aimed at improving the serum shift profile and the selectivity toward the hERG ion channel of early lead compounds **55N** and **57a**, yielding compound **60a** and **60b**. Exploration of different heterocyclic cap groups led back to the initial 7-methoxyquinoline group in combination with the methyl or ethyl substituted 6-azaspiro[2.5]octane moiety. These compounds exhibited the best potency toward HDAC1-3 and a good selectivity over HDAC6 and HDAC8, simultaneously minimizing potency toward the off-target protein hERG. Compound **60a** of the imidazole series exhibited excellent inhibitory activity below 0.4 nM toward HDAC1–3, greater than 800-fold selectivity over HDAC6 and HDAC8, and minimized potency of 42 µM toward hERG. Compound **60b** of the oxazole series was as potent and selective as **60a**, having slightly higher IC_50_ values of 0.35 nM (HDAC1), 1.3 nM (HDAC2), and <0.3 nM (HDAC3) and an IC_50_ of 34 µM toward hERG. Pharmacokinetic studies of selected compounds in rats and dogs showed a normalized clearance of 6–60 mL⋅min^−1^⋅kg^−1^, as well as a half-life of 4.8–13 h and 19–43 h in rats and dogs, for **60a** and **60b**, respectively. Noteworthy is the better bioavailability of the oxazole analog due to the improved membrane permeability. Taken together, the authors stated that compound **60b** meets the requirements for moving into further development [93]. Alternative research by Yu et al. [117] led to the discovery of arylketones as agents for HIV latency reactivation. With the aid of crystal structures, Yu and coworkers replaced the ethyl group with an aromatic ring to possibly better fit into the foot pocket and improve class I selectivity, as well as potency and physicochemical properties. Several arylketones were prepared, of which compound **61** exhibited an exceptional fit. It was suggested that sterically more demanding groups than the isoxazol result in clashes with the HDAC2 foot pocket, lowering the overall potency and, in some cases, inducing HDAC3 selectivity. Compound **61** had a slower on-kinetic of 1.82 × 10^−2^ nM⋅min^−1^ and a residence time of 35 h compared to ethylketones with on-rates of 1.7 × 10^6^ nM⋅min^−1^ and residence times in the 5 min range. The compound exhibited high total clearance of up to (156 mL·min^−1^·kg^−1^) in mice, rats, and dogs with half-lives up to 5.5 h and moderate oral bioavailability of up to 18% with a good plasma protein binding fraction of 91.5%. Inhibition of HDAC isoforms showed IC_50_ values of 0.08 nM (HDAC1), 0.47 nM (HDAC2), 0.09 nM (HDAC3), 3.0 nM (HDAC8), and over 45 µM for other isozymes [117]. Traore et al. [128] were interested in selectivity differences between class I HDACs and HDAC6 and synthesized FR23522 analogs for that purpose. They found that minimizing the structure does not significantly affect class I selectivity of the hydroxyl ketone ZBG, suggesting that the cap improves ligand potency. The best compound **63** showed inhibitory activity of 5.04 µM (HDAC1), 1.97 µM (HDAC3/NCOR2), and no significant inhibition of HDAC4 and HDAC8, as well as no inhibition of HDAC6. In silico studies of **64** suggested a bidentate and monodentate binding mode toward HDAC1 and HDAC6, respectively. Studies in cell lines showed antiproliferative activities of 2.84 µM (Jurkat), 7.12 µM (K562), 115.1 µM (Hela), and 15.9 µM (HEK293). In addition, **63** was potent toward the malaria pathogen *P. falciparum* (4.85 µM). The electrophilic trifluoromethyl ketones, originally described by Frey et al. [119], were evaluated by Gong et al. [122] via ZBG exchange using a condensed largazole scaffold [122]. The most potent compound **65** showed IC_50_ values of 26.28 nM (HDAC1), 25.84 nM (HDAC3), 31.54 nM (HDAC4), and 20.81 nM (HDAC6), as well as antiproliferative activity against MM.1S (390 nM), RPMI8226 (85 nM), NCI-H929 (1.23 µM), LP1 (370 nM), Mino (120 nM), and JeKo-1 (64 nM) cell lines. In search of selective inhibitors, Schweipert et al. [123] evaluated a series of fluorescent [1,3]dioxolo[4,5-f]benzodioxole (DBD)-based probes with a trifluoromethyl ketone warhead. The potency of the compounds increased with linker length, being the strongest for compound **66**, which inhibited HDAC isoforms with IC_50_ values of 1.0 µM (HDAC1), 4.5 µM (HDAC2), 68 nM (HDAC3), 29 nM (HDAC4), 1.0 µM (HDAC6), and 20 nM (HDAC8). Interestingly, compound **66** showed over 10-fold longer residence times than its analogs. Kinetic analysis and molecular docking suggested a two-step binding mechanism for **66**, chelating the zinc ion with the geminal diol and occupying a nearby allosteric pocket, making it selective for HDAC8 [123]. In a similar approach, Depetter et al. [129] exchanged the hydroxamate ZBG of Tubastatin A with a trifluoromethyl ketone to yield **67**. Unfortunately, the most active TFMK did not alter the acetylation status of tubulin and histone H3 and was ineffective against the SK-OV-3 cell line. These findings could be explained by the poor potency of **67** and the metabolic susceptibility, counseling appropriate caution when exchanging and comparing ZBG using the same scaffold. In summary, ketone warheads are very potent with a moderate pharmacokinetic profile. Electrophilic ketones lack inherent selectivity and display metabolic instability through glucuronidation and reduction to the equivalent alcohol [119]. Improving the metabolic stability and tuning of properties can be achieved by bulky substitutions near the ketone moiety, resulting in longer residence times and slower on-rates [117]. Strikingly, these warheads seem to be mostly used in combination with an alkyl linker, as applied in compounds **58–61**. 

## 8. Thiol Warheads

**Figure 16:**
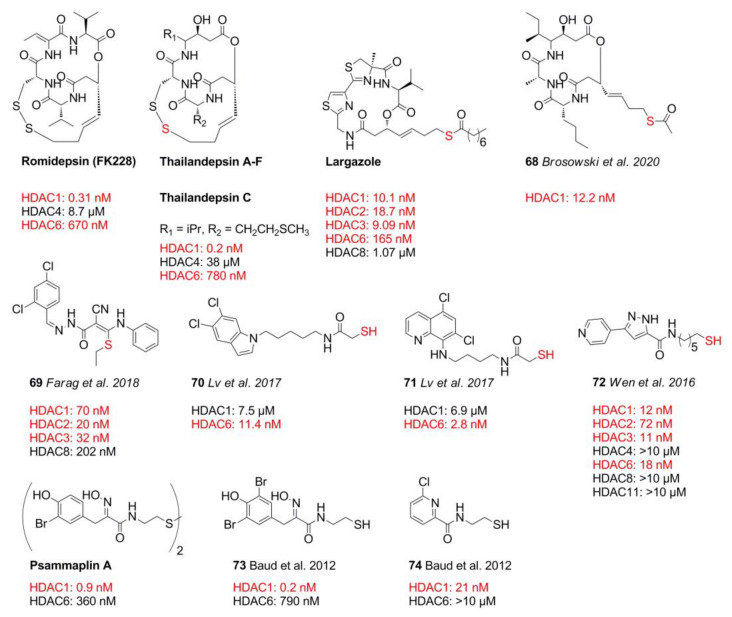
Selected sulfur-containing compounds depicted as prodrugs or in their active thiol form. Chelating atoms and IC_50_ values below 1 µM are highlighted in red.

Thiols are excellent zinc chelators and are abundant in the catalytic and structural zinc sites of proteins, indicating the thiophilic nature of zinc [130]. One of the first potent thiol-based HDACis was the natural product romidepsin (FK228) identified in 1994 [131] and approved by the FDA in 2009 [132]. Further similar natural products isolated from *Burkholderia thailandensis* by Cheng et al. [133], Biggins et al. [134], and Wang et al. [135] were thailandepsins A–F (Figure 16) with inhibitory activity in the sub nM range and improved selectivity compared to romidepsin. Largazole is another famous depsipeptide isolated from cyanobacterium *Symploca* sp. containing a thiol warhead [136]. Altogether, these disulfide prodrugs can be metabolized to their active thiol form. Unfortunately, the pharmacokinetic properties of thiols are inferior compared to other ZBGs with half-lives of under an hour [137,138]. 

Most recently, Brosowsky et al. [139] synthesized thailandepsin B pseudo-natural products with varying warheads. Compound **68** was identified as the most potent, inhibiting HDAC1 with 12.2 nM. Farag et al. [96] identified compound **69** using an in silico hopping approach. Compound **69** was synthesized and tested against class I HDACs, showing nM inhibitory activity against HDAC1 (70 nM), HDAC2 (20 nM), HDAC3 (32 nM), and HDAC8 (202 nM). Screening against the NCI-60 cell library demonstrated good inhibitory activity against HL-60 (3.2 µM) and MDA-MB-435 (420 nM) cell lines. Docking studies suggested a thioether chelating mode. A series of HDAC6 selective mercaptoacetamides was prepared by Lv et al. [138] for potential applications in CNS disorders. In contrast to prior in silico studies [140,141], which suggested a bidentate binding mode, crystal structure data of drHDAC6 and smHDAC8 with a mercaptoacetamide ZBG showed a monodentate coordination by the thiol group [39,142]. The most potent compounds **70** and **71** inhibited HDAC6 with 11.4 nM and 2.8 nM and had 600- to 2400-fold selectivity over HDAC1. As expected, their ester and disulfide produgs were more potent against HEK cells and triggered tubulin acetylation. The general short half-life time of disulfides is to be noted [137]. Half-life times of the disulfide prodrugs consisting of **70** and **71** moieties were measured in the range of 20.7–49.5 min in human liver microsomes [138]. In 2016, Wen et al. [143] synthesized pyrazole-containing thiol compounds with class I and IIb preference. The most potent compound **72** had an inhibitory activity in the range of 11–72 nM toward HDAC1–3 and HDAC6 [143]. Regarding in vivo studies, the authors noted that their disulfide compounds were unexpectedly 2–8-fold more potent that their thioester counterparts. Antiproliferative activities were determined against HCT-116 (8.93 µM), HT-29 (6.92 µM) MCF-7 (7.15 µM), MDA-MB-231 (27.31 µM), A549 (6.07 µM), PC-3 (4.74 µM), and AsPC-1 (25.31 µM) cell lines. The control HEK-293 cell line with an GI_50_ of 14.77 µM suggested moderate cytotoxicity. Noteworthy are the studies by Baud et al. [144], in which the binding mode of psammaplin A, a marine metabolite, was validated and studied. For this purpose, a series of compounds were prepared, whereby it was demonstrated in several complementary assays that psammaplin A acts as an isozyme-selective thiol-ZBG prodrug with exceptional inhibitory activity of 0.9 nM toward HDAC1 and 400-fold selectivity over HDAC6. Further investigations of the linker moiety showed that the oxime moiety was crucial for HDAC1 potency and selectivity. An evaluation of the cap group led to **73**, which showed outstanding 3950-fold selectivity over HDAC6 with an inhibitory activity of 0.2 nM. Evaluation against A549, MCF7, and WI38 cancer cell lines showed GI_50_ values of 2.53 µM, 2.35 µM, and 3.4 µM, respectively. Follow-up studies [145] were aimed at the replacement of the oxime group with an aryl moiety and yielded compound **74**. The superior potency of six-membered nitrogen containing aryls compared to five-membered heterocycles could be further amplified by chlorine substitution, showing a potency of 21 nM and over 470-fold selectivity for HDAC1. To sum up, thiol warheads are excellent warheads with sub nM inhibition potencies, but suffer from metabolic instability, although romidepsin is approved by FDA. Nevertheless, these are valuable compounds in terms of SAR exploration and as model compounds under assay conditions.

## 9. Carboxylic Acids

**Figure 17:**
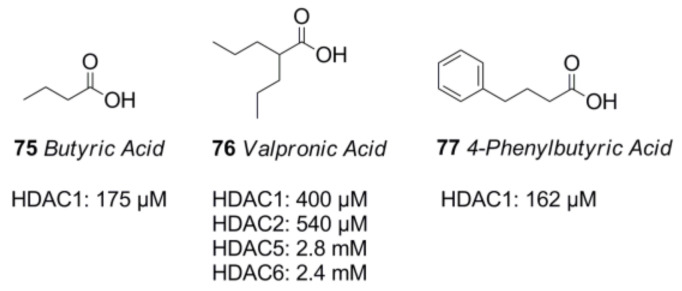
Most famous acid derivatives used in earlier studies.

Carboxylic acids such as butyric acid **75**, valpronic acid **76**, or 4-phenylbutyric acid **77** usually exhibit inhibitory activity in the mM range and play only a negligible role in the field of pharmaceutical inhibitors [146,147,148,149] (Figure 17). Worth mentioning in this context is the favorable effect of a ketogenic diet, producing fatty acids as the main cell fuel yielding positive effects in the fields of longevity [150] and anti-inflammation [151], as well as reducing oxidative stress [152], partially through HDAC inhibition. 

## 10. Trifluoromethyloxadiazole (TFMO) Warheads

**Figure 18:**
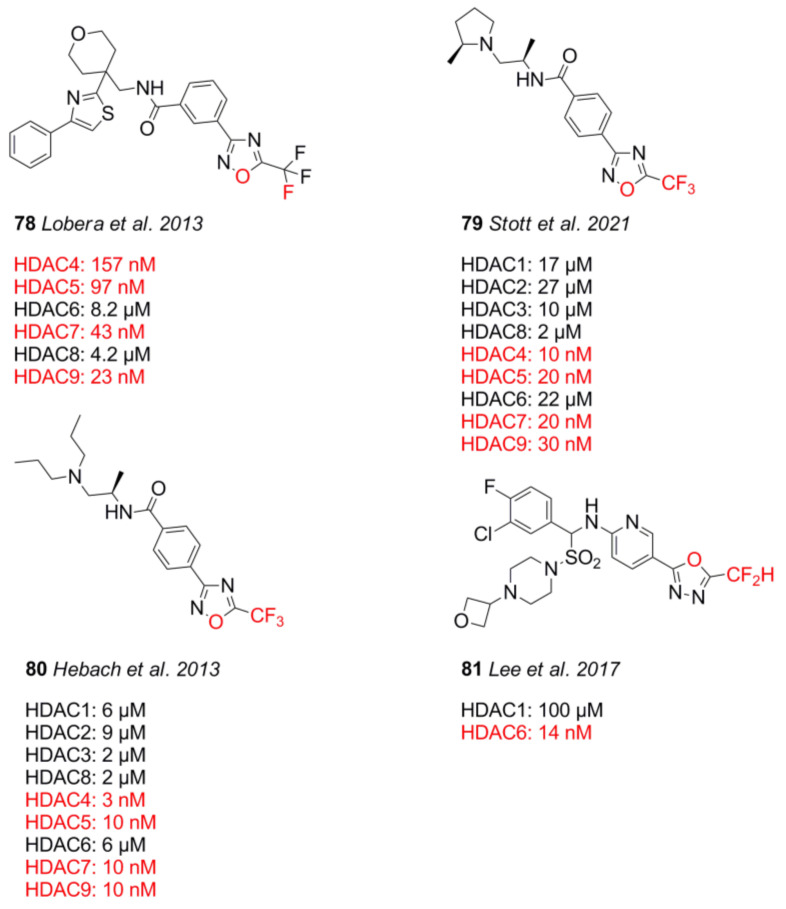
A selection of potent TFMO-containing compounds. Chelating atoms are exemplified in **78** and highlighted in red for IC_50_ values below 1 µM.

Another class of ZBGs consists of heterocycles, which can be branched according to their core motif. Five-membered azoles seem to be non-chelating ZBGs with inhibitory activity in the nM range and tunable selectivity toward class IIa and b, (Figure 18). The TFMO warhead interacts with the zinc ion via the oxygen in the oxazole moiety and a fluorine atom with distances of 3.0 Å and 2.7 Å, respectively [153]. 

In 2013, Lobera et al. [153] identified the non-chelating trifluoromethyloxadiazole ZBG. Compound **78** had preference for class IIa HDACs and inhibited recombinant isoforms with 157 nM (HDAC4), 97 nM (HDAC5), 43 nM (HDAC7), and 23 nM (HDAC9), as well as 8.2 µM (HDAC6) and 4.2 µM (HDAC8). Most recently, inspired by the work of Lobera et al. [153] and Guerriero et al. [154], Stott et al. [155] searched for CNS-penetrant class IIb HDACis and identified the class IIb-selective compound **79** for use in preclinical models of Huntington’s disease. Starting from compound **80** from Hebach et al. [156], an SAR study was conducted by derivatizing the TFMO group, the linker, and the cap group [156]. The authors confirmed that the trifluoromethyl group, as well as the position of the oxygen in the TFMO moiety, was crucial for potency. Stability assessments of the electron-deficient TFMO group showed no degradation as probed by NMR in DMSO-d6 for 6 days. The poor metabolic stability of **80** caused by the lipophilic basic amine moiety was overcome by substitution with a terminal pyrrolidine group, which reduced overall lipophilicity by a factor of 0.6. lastly, a linker screening identified an (*R*)-methyl substituent as a suitable building block with a threefold improvement in inhibitory activity against HDAC4. Inhibitory activity of **79** against HDAC isozymes was determined to be 10 nM (HDAC4), 20 nM (HDAC5), 20 nM (HDAC7), and 30 nM (HDAC9) for class IIa, 17 µM (HDAC1), 27 µM (HDAC2), 10 µM (HDAC3), and 2.0 µM (HDAC8) for class I, and 22 µM for HDAC6. Additional cell studies showed an inhibitory activity of 40 nM and 10 nM toward Jurkat and HEK293 cells, respectively. Compound **79** demonstrated overall acceptable pharmacokinetic properties, and it was stable in mouse and human plasma and blood, as well as simulated gastric fluid, showing a distribution ratio of 3:1 blood to plasma with an unbound fraction of 0.73 and 0.17 in blood and brain homogenate, respectively. It showed 100% oral bioavailability and reached a maximum concentration in blood and brain after just 0.5 h. The compound had a high distribution volume of 3.8 L⋅kg^−1^ and a high brain-to-blood exposure ratio of up to 3, but a high plasma clearance of 4.2 L⋅h^−1^⋅kg^−1^. An off-target assessment was undertaken with a variety of assays, exhibiting no inhibition of cytochrome P450, an inhibitory activity of 3.9 µM toward hERG, up to 80% inhibition of the sigma-1 receptor, and an inhibition of greater than 80% toward muscarinic M1–M5 receptors. Worth mentioning are also 1,3,4-oxadiazole compounds by Lee et al. [157], which showed HDAC6 preference. Compound **81** inhibited HDAC6 with an IC_50_ value of 14 nM and showed 7142-fold selectivity over HDAC1. TFMO warheads are unique non-chelating ZBGs and achieve their affinity through coordination with the oxygen and fluorine atom. This fact allows exploitation for class II selectivity and potency. Additional good pharmacokinetics, as well as the metabolic stability, makes this ZBG an attractive scaffold for further development. 

## 11. Thiazolidinedione (TZD) Warheads

**Figure 19:**
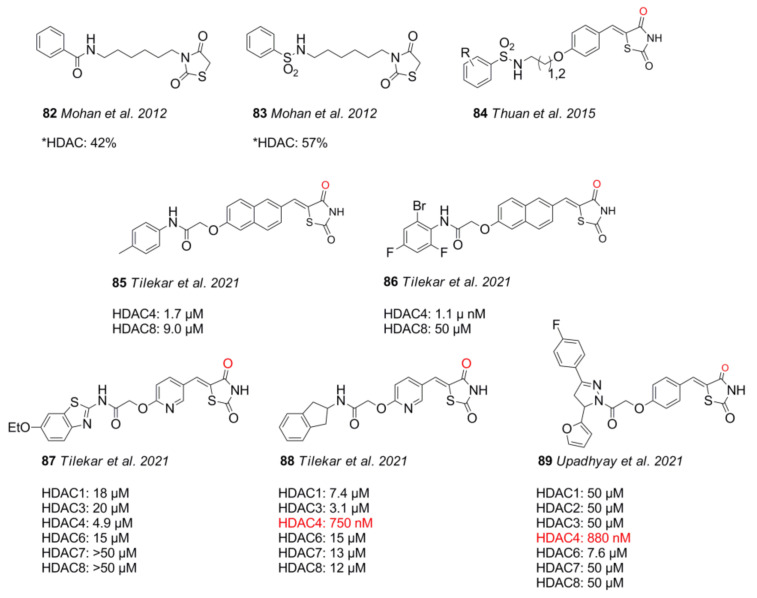
TZD-containing HDACis. Proposed chelating atoms are highlighted in red, as along with IC_50_ values below 1 µM. * Inhibitory potential is given as the percentage inhibition of HDACs in HepG2 nuclear extract in the presence of 100 µM **82** or **83**.

Another type of non-hydroxamate HDACi is based on the thiazolidine dione ring, (Figure 19). Some thiazolidine dione-containing substances have been approved for diabetes type II treatment, and they exhibit manifold biological activities with effects depending on compound, species, cell, and concentration [158].

In 2012, Mohan et al. tested TZD-SAHA analogs against the HepG2 liver cancer cell line showing 42–57% cell death after incubation with 100 µM of **82** and **83** for 48 h. HDAC assays using nuclear extracts as an HDAC source showed maximum HDAC inhibition in the 8 h interval and comparable inhibition of **82** and **83** to positive control SAHA [98]. In an effort to synthesize PTP1B inhibitors, Thuan et al. synthesized analogs of **84**, which had comparable cytotoxicities in colon, prostate, and lung cancer cell lines (SW620, PC-3, NCI-H460) to SAHA as control but were not potent against the protein of interest. Evaluation of histone acetylation and the previous literature [98] indicated a potential to inhibit HDACs [159]. Most recently, Tilekar et al. [97] and Upadhyaya et al. [99] designed compounds **32–34** (Figure 7) with the intention of using the TZD group as ZBG. Further investigations by Tilekar et al. [160] with the objective of designing dual HDAC4, HDAC8, and PPARγ inhibitors led to exemplary compound **85** and **86**, which differ in the substitution pattern of the naphthyl linker compared to **32–34**, forming a more extended shape. In silico analysis supported a complexation via the TZD group in HDAC4. Compound **85** exhibited an inhibitory activity of 1.7 µM against HDAC4 and an EC_50_ value of 245 nM against PPARγ. Evaluation of **86** showed an IC_50_ of 1.1 µM toward HDAC4, yielded cell apoptosis in several cancer cell lines, and caused DNA fragmentation in CEM cells with an IC_50_ value of 9.6 µM. Additional evaluation of **85** in CCRF-CEM tumor xenografts led to significant tumor regression [160]. Follow-up studies by Tilekar et al. [161] identified TZD derivatives with a pyridine linker, replacing the naphthyl group. The most potent compound of the series **87** showed a potency of 4.9 µM toward HDAC4 and greater than 10-fold selectivity over HDAC8. Compound **87** was most effective against lymphoblastic leukemia (CCRF-CEM) cell lines with a CC_50_ value of 15.2 µM, inhibiting HeLa and MDA-MB-231 with 10.5 µM and 10.1 µM, respectively. The authors attributed the preference to bind HDAC4 to the carbonyl group of the TZD warhead. It was noted that previously described N-substituted TZD analogs were inactive, highlighting the importance of the NH group for HDAC4 selectivity [161]. Interestingly, compound **88** showed inhibitory activity of 750 nM and 12 µM toward HDAC4 and HDAC8, respectively, supporting prior studies suggesting a chelation of HDAC8 via the amide carbonyl. Similar studies by Upadhyay et al. [162] identified **89** as a dual inhibitor of HDAC and vascular endothelial growth factor 2, (VEGFR-2), confirmed by inhibiting HUVEC cell proliferation, migration, and tube formation in vitro. Compound **89** showed an IC_50_ of 880 nM toward HDAC4 and greater than 50-fold selectivity over HDAC8. Evaluation in cancer cell lines showed IC_50_ values of 28.41 µM (MCF-7), 46.27 µM (K562), 19.52 µM (A549), and 18.84 µM (HT-29). Noteworthily, in silico studies indicated further interactions of the amide–pi stacking with G811, pi–sulfur interactions with H842, and pi–alkyl interactions with L943, in addition to chelation of zinc via the carbonyl group of the TZD warhead. TZD warheads, albeit moderately potent, show very promising potency and selectivity trends. The additional inhibition of other targets such as PPAR-γ and VEGFR-2 makes them promising candidates for future clinical applications. 

## 12. Carbamate Scaffold Warheads

**Figure 20:**
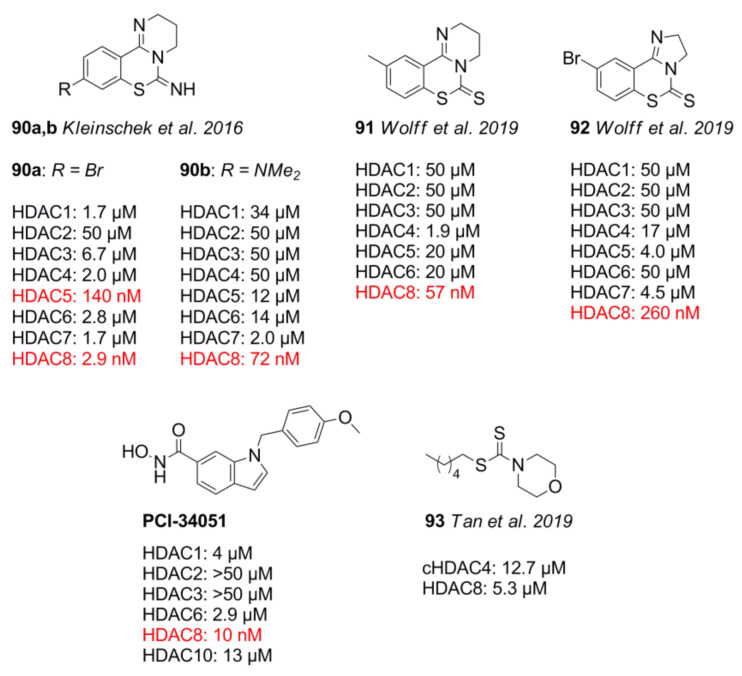
Diverse thiocarbamate-containing scaffolds. Activities below 1 µM are highlighted in red.

An unusual 1,3-benzothiazine scaffold was described by Kleinschek et al. [163], which had beneficial starting properties for further optimization. An SAR study showed preference for the imine moiety, larger ring sizes, and tolerance for substitution at aromatic positions with small groups as in **90a** but not with a dimethyl amino group as in **90b**, which decreased potency more than 24-fold (Figure 20). Model compound **90a** was tested against HDAC isoforms with inhibitory activity of 1.7 µM (HDAC1), >50 µM (HDAC2), 6.7 µM (HDAC3), 2.0 µM (HDAC4), 140 nM (HDAC5), 2.8 µM (HDAC6), 1.7 µM (HDAC7), and 2.9 nM (HDAC8), and it exhibited GI_50_ values in the range of 11–1000 µM against SK-UT-1, MCF7, and Jurkat cell lines depending on the substitution pattern. In a follow-up study, Muth et al. showed that the parent compound of **90a** lacking a bromine substituent was decomposed in the presence of intracellular GSH and acted as a covalent modifier of HDAC8 [164]. In an effort to improve the chemical stability of **90a** and analogs thereof, Wolff et al. developed thione derivatives with compound **91** being the most potent inhibitor for HDAC8 (IC_50_ = 57 nM) and showing outstanding isozyme selectivity [165]. 1,3-Benzothiazine analogs were shown to be extremely robust against mM concentrations of GSH and able to penetrate cell membranes. Compound **92** reduced the proliferation of SK-N-BE(2)-C neuroblastoma cells in the µM range, similarly to the reference compound PCI-34051 [166]. Intracellular inhibition of HDAC8 was demonstrated by increased acetylation of the specific bona fide substrate acetyl-SMC3 and led to a patent [167]. A similar noncyclic dithiocarbamate **93** was recently reported by Tan et al. [165] Compound analogs of **93** were tested against HDAC8 and cHDAC4, showing medium to weak inhibition in the range of 6–45 µM. Since **93** is almost the size of a fragment, this chemically easily accessible scaffold holds promise as a starting point for the development of more potent HDACis lacking traditional ZBGs. The presented compounds show very potent and selective inhibition profiles with low molecular weight, making them an attractive starting point for further development.

## 13. Miscellaneous

**Figure 21:**
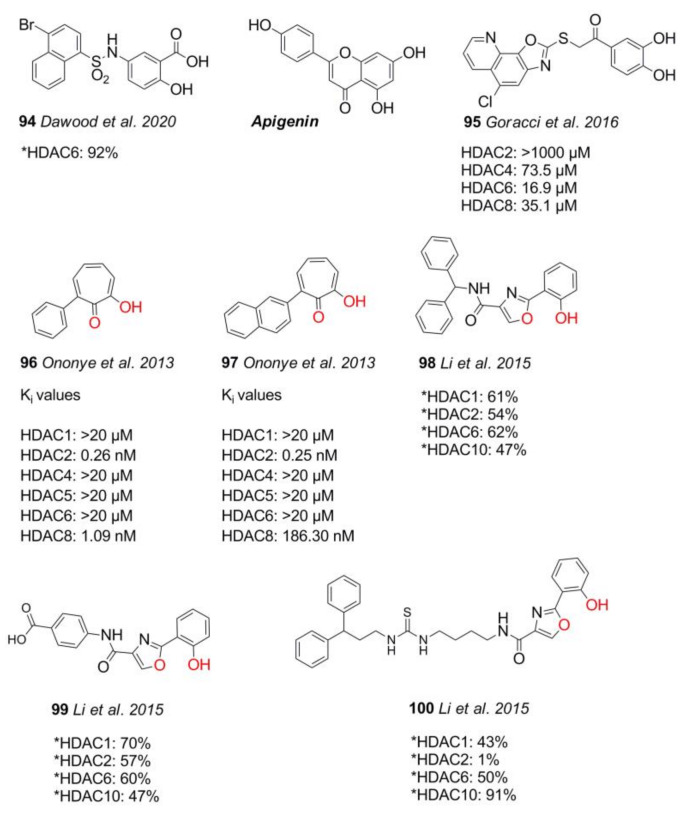
Shown are miscellaneous compounds with diverse ZBGs. Ononye et al. [170] and Li et al. [172], through docking, suggested the chelating atoms highlighted in red. * Inhibitory activity is given as the percentage inhibition of HDACs in the presence of **94** (10 µM) and **98–100** (20 µM). K_i_ values are displayed for compounds **96** and **97**.

Recently, Dawood et al. [168] identified a hydroxybenzoic acid-based compound **94** following virtual screening of two databases. Compound **94** was tested against four cell lines and showed antiproliferative activities in the range of 55 µM and 73 µM toward sensitive and multidrug-resistant cell lines CCRF-CEM and CEM/ADR5000, respectively. In 2012, Pandey et al. [169] conducted an in vivo study in xenograft prostate cancer models, and they showed a reduction in tumor growth with an intake of 20 and 50 µg/mouse/day of apigenin. This was associated with increased histone acetylation, histone-3 (H3) hyperacetylation on the p21/waf1 promoter, and a reduction in HDAC activity and expression. Catechol derivative **95** was synthesized and tested by Goracci et al. [112], exhibiting inhibitory activity in the µM range with selectivity toward HDAC6 and HDAC8. As compound **95** had only twofold selectivity toward HDAC6, it was not considered for further studies. Ononye et al. [170] synthesized highly HDAC2 selective tropolone derivatives with inhibitory activities in the nM range. Substitution analysis showed preference for large residues in the beta position, which could be rationalized by docking studies. Derivatization and competition experiments showed active site-based activity, and they depicted the hydroxyl as an important factor for inhibition. On the basis of the low molecular weight, the metal-binding properties, and opportunities for diversification, the tropolone scaffold was considered drug-like and suggested for further development. One potent compound (**96**) exhibited a K_i_ value of 1.09 nM (HDAC8) and was further tested against hematological and solid tumor cell lines. Several compounds were effective in T lymphocyte cell lines, showing GI_50_ values below 1 µM, but less activity against HCT-116 and BxPC-3 cells, with values in the range of 14–180 µM. Pharmacokinetic assessment showed a half-life time of 93 min in mouse liver microsomes, as well as potential for glucuronidation, but no general cytotoxicity against human dermal fibroblast cells [170]. Further studies by Haney et al. [171] identified **97**, which showed antiproliferative activities in the range of 1–11 µM against three different myeloma cell lines and exhibited a different apoptosis mechanism as a function of gene expression patterns compared to SAHA. An oxazole-based compound **98** was developed by Li and Woster [172] in an attempt to structurally mimic the benzamide ZBG and simultaneously reduce the potential metabolic toxicity. Evaluation of zinc-binding affinity using ITC by titration of compounds to zinc chloride resulted in a ΔG of −21.35 kJ⋅mol^−1^ for **98** and ΔG = −15.0 kJ⋅mol^−1^ for SAHA, indicating the more efficient binding of compound **98**. These oxazole-based compounds exhibited mostly negligible cap group-based preferences toward tested HDAC1, HDAC6, and HDAC10 and did not inhibit other recombinant isozymes over 50% at 20 µM concentration. Compounds **99** and **100** in Figure 21 were most potent toward HDAC1 and HDAC10. Evaluation of **98** in the MV-4-11 leukemia cell line showed an IC_50_ of 7.5 µM, inducing significantly increased histone acetylation (H3K9) and inhibition via p21^WAF1/CIP1^ activation. It is worth mentioning the improved performance in cell assays compared to recombinant HDACs, indicating a second mechanism of action. 

## 14. Conclusions

Due to the broad functional diversity of HDACs, these enzymes have become promising versatile targets in areas as diverse as cancer, neurodegenerative diseases, diabetes, obesity, inflammation, and even antiviral applications, e.g., via HIV latency reversal. Currently, five HDACis, vorinostat, romidepsin, belinostat, panobinostat, and chidamide, are approved for therapeutic intervention. The most successful clinical indications are hematological neoplasms and multiple myeloma. Hydroxamic acids constitute by far the largest group of HDACis due to their pronounced capability to form high-affinity chelates with the catalytic zinc ion at the bottom of the active site. However, hydroxamates have a propensity for nonspecificity and have recently come under considerable suspicion because of potential mutagenicity. Therefore, there are significant concerns when applying hydroxamate-containing compounds as therapeutics in chronic diseases beyond oncology due to unwanted toxic side effects. Consequently, considerable effort was undertaken to develop alternative ZBGs to replace the critical hydroxamate group in HDACis, while preserving high potency. This review provides an overview about recent developments toward potent and selective HDACis lacking the widely represented hydroxamate ZBG. Canonical HDACis consist of three parts: a ZBG, a linker, and a cap group. Typically, most of the affinity is conferred by the ZBG, and the cap group is used to create selectivity against other metalloproteins, particularly HDAC isozymes. Replacing high-affinity ZBGs such as hydroxamate or thiol with other functionalities usually leads to a drop in potency. Therefore, the development of potent non-hydroxamate HDACis implies that the cap group and/or linker has to make a substantial contribution to affinity in order to compensate for losses due to weaker zinc binding. On the other hand, novel ZBGs offer great potential for improving selectivity. For example, the selectivity of benzamide HDACis can be tuned by suitable substitutions at the ZBG. Furthermore, depending on the structural and dynamic differences between HDAC isozymes, especially with regard to static and transient selectivity pockets, not only the ZBG and cap group, but also the length, bulkiness, and shape, e.g., linear versus L-shaped, have a dramatic impact on selectivity. This is in contrast to the traditional design of HDACis, where the ZBG is supposed to confer affinity, and the cap group is used to tune selectivity through contacts with the protein surface. Thus, turning away from the canonical hydroxamate ZBG led to a paradigm shift from the traditional approach of inhibitor design toward a concept where all parts of the active compound (ZBG, linker, and cap group) together contribute to both affinity and selectivity. It is noteworthy that some non-hydroxamate HDACis have evolved, where the classic three-way division (ZBG, linker, and cap) has vanished and transformed into a more or less compact structure with shared functionalities. It is anticipated that the trend toward non-hydroxamate HDACis is likely to become more pronounced, and it will eventually result in an increasing number of clinical non-hydroxamate HDACi candidates with improved safety profiles, which open new perspectives for the treatment of chronic and noncancer diseases.

## Data Availability

Not applicable.

## References

[1] Ropero S., Esteller M. (2007). The Role of Histone Deacetylases (HDACs) in Human Cancer. Mol. Oncol..

[2] Kim G.-W., Yang X.-J. (2011). Comprehensive Lysine Acetylomes Emerging from Bacteria to Humans. Trends Biochem. Sci..

[3] Tessarz P., Kouzarides T. (2014). Histone Core Modifications Regulating Nucleosome Structure and Dynamics. Nat. Rev. Mol. Cell Biol..

[4] Sambucetti L.C., Fischer D.D., Zabludoff S., Kwon P.O., Chamberlin H., Trogani N., Xu H., Cohen D. (1999). Histone Deacetylase Inhibition Selectively Alters the Activity and Expression of Cell Cycle Proteins Leading to Specific Chromatin Acetylation and Antiproliferative Effects. J. Biol. Chem..

[5] Hirose T., Sowa Y., Takahashi S., Saito S., Yasuda C., Shindo N., Furuichi K., Sakai T. (2003). P53-Independent Induction of Gadd45 by Histone Deacetylase Inhibitor: Coordinate Regulation by Transcription Factors Oct-1 and NF-Y. Oncogene.

[6] Klisovic D.D., Katz S.E., Effron D., Klisovic M.I., Wickham J., Parthun M.R., Guimond M., Marcucci G. (2003). Depsipeptide (FR901228) Inhibits Proliferation and Induces Apoptosis in Primary and Metastatic Human Uveal Melanoma Cell Lines. Investig. Ophthalmol. Vis. Sci..

[7] Eckschlager T., Plch J., Stiborova M., Hrabeta J. (2017). Histone Deacetylase Inhibitors as Anticancer Drugs. Int. J. Mol. Sci..

[8] Xu W.S., Parmigiani R.B., Marks P.A. (2007). Histone Deacetylase Inhibitors: Molecular Mechanisms of Action. Oncogene.

[9] Mrakovcic M., Kleinheinz J., Fröhlich L.F. (2019). P53 at the Crossroads between Different Types of HDAC Inhibitor-Mediated Cancer Cell Death. Int. J. Mol. Sci..

[10] Ho T.C.S., Chan A.H.Y., Ganesan A. (2020). Thirty Years of HDAC Inhibitors: 2020 Insight and Hindsight. J. Med. Chem..

[11] McClure J.J., Inks E.S., Zhang C., Peterson Y.K., Li J., Chundru K., Lee B., Buchanan A., Miao S., Chou C.J. (2017). Comparison of the Deacylase and Deacetylase Activity of Zinc-Dependent HDACs. ACS Chem. Biol..

[12] Ito A., Kawaguchi Y., Lai C.-H., Kovacs J.J., Higashimoto Y., Appella E., Yao T.-P. (2002). MDM2—HDAC1-Mediated Deacetylation of P53 Is Required for Its Degradation. EMBO J..

[13] Martínez-Balbás M.A., Bauer U.-M., Nielsen S.J., Brehm A., Kouzarides T. (2000). Regulation of E2F1 Activity by Acetylation. EMBO J..

[14] Gaughan L., Logan I.R., Cook S., Neal D.E., Robson C.N. (2002). Tip60 and Histone Deacetylase 1 Regulate Androgen Receptor Activity through Changes to the Acetylation Status of the Receptor. J. Biol. Chem..

[15] Dowling D.P., Di Costanzo L., Gennadios H.A., Christianson D.W. (2008). Evolution of the Arginase Fold and Functional Diversity. Cell. Mol. Life Sci..

[16] Lombardi P.M., Cole K.E., Dowling D.P., Christianson D.W. (2011). Structure, Mechanism, and Inhibition of Histone Deacetylases and Related Metalloenzymes. Curr. Opin. Struct. Biol..

[17] Micelli C., Rastelli G. (2015). Histone Deacetylases: Structural Determinants of Inhibitor Selectivity. Drug Discov. Today.

[18] Haberland M., Montgomery R.L., Olson E.N. (2009). The Many Roles of Histone Deacetylases in Development and Physiology: Implications for Disease and Therapy. Nat. Rev. Genet..

[19] Prior R., Van Helleputte L., Klingl Y.E., Van Den Bosch L. (2018). HDAC6 as a Potential Therapeutic Target for Peripheral Nerve Disorders. Expert Opin. Ther. Targets.

[20] Mazzocchi M., Collins L.M., Sullivan A.M., O’Keeffe G.W. (2020). The Class II Histone Deacetylases as Therapeutic Targets for Parkinson’s Disease. Neuronal Signal..

[21] Duvic M., Vu J. (2007). Vorinostat in Cutaneous T-Cell Lymphoma. Drugs Today.

[22] Campàs-Moya C. (2009). Romidepsin for the Treatment of Cutaneous T-Cell Lymphoma. Drugs Today.

[23] Lee H.-Z., Kwitkowski V.E., Del Valle P.L., Ricci M.S., Saber H., Habtemariam B.A., Bullock J., Bloomquist E., Shen Y.L., Chen X.-H. (2015). FDA Approval: Belinostat for the Treatment of Patients with Relapsed or Refractory Peripheral T-Cell Lymphoma. Clin. Cancer Res..

[24] Raedler L.A. (2016). Farydak (Panobinostat): First HDAC Inhibitor Approved for Patients with Relapsed Multiple Myeloma. Am. Health Drug Benefits.

[25] Lu X., Ning Z., Li Z., Cao H., Wang X. (2016). Development of Chidamide for Peripheral T-Cell Lymphoma, the First Orphan Drug Approved in China. Intractable Rare Dis. Res..

[26] Finnin M.S., Donigian J.R., Cohen A., Richon V.M., Rifkind R.A., Marks P.A., Breslow R., Pavletich N.P. (1999). Structures of a Histone Deacetylase Homologue Bound to the TSA and SAHA Inhibitors. Nature.

[27] Corminboeuf C., Hu P., Tuckerman M.E., Zhang Y. (2006). Unexpected Deacetylation Mechanism Suggested by a Density Functional Theory QM/MM Study of Histone-Deacetylase-Like Protein. J. Am. Chem. Soc..

[28] Gantt S.L., Joseph C.G., Fierke C.A. (2010). Activation and Inhibition of Histone Deacetylase 8 by Monovalent Cations. J. Biol. Chem..

[29] Vanommeslaeghe K., Proft F.D., Loverix S., Tourwé D., Geerlings P. (2005). Theoretical Study Revealing the Functioning of a Novel Combination of Catalytic Motifs in Histone Deacetylase. Bioorg. Med. Chem..

[30] Lauffer B.E.L., Mintzer R., Fong R., Mukund S., Tam C., Zilberleyb I., Flicke B., Ritscher A., Fedorowicz G., Vallero R. (2013). Histone Deacetylase (HDAC) Inhibitor Kinetic Rate Constants Correlate with Cellular Histone Acetylation but Not Transcription and Cell Viability. J. Biol. Chem..

[31] Somoza J.R., Skene R.J., Katz B.A., Mol C., Ho J.D., Jennings A.J., Luong C., Arvai A., Buggy J.J., Chi E. (2004). Structural Snapshots of Human HDAC8 Provide Insights into the Class I Histone Deacetylases. Structure.

[32] Shen S., Kozikowski A.P. (2016). Why Hydroxamates May Not Be the Best Histone Deacetylase Inhibitors—What Some May Have Forgotten or Would Rather Forget?. ChemMedChem.

[33] McClure J.J., Li X., Chou C.J. (2018). Advances and Challenges of HDAC Inhibitors in Cancer Therapeutics. Adv. Cancer Res..

[34] Eom H., Song W.J. (2019). Emergence of Metal Selectivity and Promiscuity in Metalloenzymes. J. Biol. Inorg. Chem..

[35] Laitaoja M., Valjakka J., Jan J. (2013). Zinc Coordination Spheres in Protein Structures. Inorg. Chem..

[36] Wang S. (2020). Single Molecule Observation of Hard–Soft-Acid–Base (HSAB) Interaction in Engineered Mycobacterium Smegmatis Porin A (MspA) Nanopores. Chem. Sci..

[37] Christianson D.W. (1991). Structural Biology of Zinc. Adv. Protein Chem..

[38] Dowling D.P., Gantt S.L., Gattis S.G., Fierke C.A., Christianson D.W. (2008). Structural Studies of Human Histone Deacetylase 8 and Its Site-Specific Variants Complexed with Substrate and Inhibitors. Biochemistry.

[39] Stolfa D.A., Marek M., Lancelot J., Hauser A.-T., Walter A., Leproult E., Melesina J., Rumpf T., Wurtz J.-M., Cavarelli J. (2014). Molecular Basis for the Antiparasitic Activity of a Mercaptoacetamide Derivative That Inhibits Histone Deacetylase 8 (HDAC8) from the Human Pathogen Schistosoma Mansoni. J. Mol. Biol..

[40] Wang C.Y. (1977). Mutagenicity of Hydroxamic Acids for Salmonella Typhimurium. Mutat Res. Fund Mol. Mech. Mutagen..

[41] Zhao C., Dong H., Xu Q., Zhang Y. (2020). Histone Deacetylase (HDAC) Inhibitors in Cancer: A Patent Review (2017–Present). Expert Opin. Ther. Pat..

[42] Melesina J., Simoben C.V., Praetorius L., Bülbül E.F., Robaa D., Sippl W. (2021). Strategies To Design Selective Histone Deacetylase Inhibitors. ChemMedChem.

[43] Meyners C., Meyer-Almes F.-J. (2017). Impact of Binding Mechanism on Selective Inhibition of Histone Deacetylase Isoforms. Chem. Biol. Drug Des..

[44] Yung-Chi C., Prusoff W.H. (1973). Relationship between the Inhibition Constant (KI) and the Concentration of Inhibitor Which Causes 50 per Cent Inhibition (I50) of an Enzymatic Reaction. Biochem. Pharmacol..

[45] Meyners C., Baud M.G.J., Fuchter M.J., Meyer-Almes F.-J. (2014). Kinetic Method for the Large-Scale Analysis of the Binding Mechanism of Histone Deacetylase Inhibitors. Anal. Biochem..

[46] Géraldy M., Morgen M., Sehr P., Steimbach R.R., Moi D., Ridinger J., Oehme I., Witt O., Malz M., Nogueira M.S. (2019). Selective Inhibition of Histone Deacetylase 10: Hydrogen Bonding to the Gatekeeper Residue Is Implicated. J. Med. Chem..

[47] Meyners C., Mertens M., Wessig P., Meyer-Almes F.-J. (2017). A Fluorescence-Lifetime-Based Binding Assay for Class IIa Histone Deacetylases. Chem. Eur. J..

[48] Riester D., Hildmann C., Haus P., Galetovic A., Schober A., Schwienhorst A., Meyer-Almes F.-J. (2009). Non-Isotopic Dual Parameter Competition Assay Suitable for High-Throughput Screening of Histone Deacetylases. Bioorg. Med. Chem. Lett..

[49] Matulis D., Kranz J.K., Salemme F.R., Todd M.J. (2005). Thermodynamic Stability of Carbonic Anhydrase: Measurements of Binding Affinity and Stoichiometry Using ThermoFluor. Biochemistry.

[50] Robers M.B., Dart M.L., Woodroofe C.C., Zimprich C.A., Kirkland T.A., Machleidt T., Kupcho K.R., Levin S., Hartnett J.R., Zimmerman K. (2015). Target Engagement and Drug Residence Time Can Be Observed in Living Cells with BRET. Nat. Commun..

[51] Hubbert C., Guardiola A., Shao R., Kawaguchi Y., Ito A., Nixon A., Yoshida M., Wang X.-F., Yao T.-P. (2002). HDAC6 Is a Microtubule-Associated Deacetylase. Nature.

[52] Dasgupta T., Antony J., Braithwaite A.W., Horsfield J.A. (2016). HDAC8 Inhibition Blocks SMC3 Deacetylation and Delays Cell Cycle Progression without Affecting Cohesin-Dependent Transcription in MCF7 Cancer Cells. J. Biol. Chem..

[53] Saito A., Yamashita T., Mariko Y., Nosaka Y., Tsuchiya K., Ando T., Suzuki T., Tsuruo T., Nakanishi O. (1999). A Synthetic Inhibitor of Histone Deacetylase, MS-27-275, with Marked in Vivo Antitumor Activity against Human Tumors. Proc. Natl. Acad. Sci. USA.

[54] Suzuki T., Ando T., Tsuchiya K., Fukazawa N., Saito A., Mariko Y., Yamashita T., Nakanishi O. (1999). Synthesis and Histone Deacetylase Inhibitory Activity of New Benzamide Derivatives. J. Med. Chem..

[55] Sangwan R., Rajan R., Mandal P.K. (2018). HDAC as Onco Target: Reviewing the Synthetic Approaches with SAR Study of Their Inhibitors. Eur. J. Med. Chem..

[56] Ning Z.-Q., Li Z.-B., Newman M.J., Shan S., Wang X.-H., Pan D.-S., Zhang J., Dong M., Du X., Lu X.-P. (2012). Chidamide (CS055/HBI-8000): A New Histone Deacetylase Inhibitor of the Benzamide Class with Antitumor Activity and the Ability to Enhance Immune Cell-Mediated Tumor Cell Cytotoxicity. Cancer Chemother. Pharmacol..

[57] Zhang L., Zhang J., Jiang Q., Zhang L., Song W. (2018). Zinc Binding Groups for Histone Deacetylase Inhibitors. J. Enzym. Inhib. Med. Chem..

[58] Marson C.M., Matthews C.J., Atkinson S.J., Lamadema N., Thomas N.S.B. (2015). Potent and Selective Inhibitors of Histone Deacetylase-3 Containing Chiral Oxazoline Capping Groups and a *N*-(2-Aminophenyl)-Benzamide Binding Unit. J. Med. Chem..

[59] Chou C.J., Herman D., Gottesfeld J.M. (2008). Pimelic Diphenylamide 106 Is a Slow, Tight-Binding Inhibitor of Class I Histone Deacetylases. J. Biol. Chem..

[60] Fraczek J., Vanhaecke T., Rogiers V. (2013). Toxicological and Metabolic Considerations for Histone Deacetylase Inhibitors. Expert Opin. Drug Metab. Toxicol..

[61] Benigni R., Passerini L. (2002). Carcinogenicity of the Aromatic Amines: From Structure–Activity Relationships to Mechanisms of Action and Risk Assessment. Mutat. Res..

[62] Benigni R., Passerini L., Gallo G., Giorgi F., Cotta-Ramusino M. (1998). QSAR Models for Discriminating between Mutagenic and Nonmutagenic Aromatic and Heteroaromatic Amines. Environ. Mol. Mutagen..

[63] Beconi M., Aziz O., Matthews K., Moumné L., O’Connell C., Yates D., Clifton S., Pett H., Vann J., Crowley L. (2012). Oral Administration of the Pimelic Diphenylamide HDAC Inhibitor HDACi 4b Is Unsuitable for Chronic Inhibition of HDAC Activity in the CNS In Vivo. PLoS ONE.

[64] Ryan Q.C., Headlee D., Acharya M., Sparreboom A., Trepel J.B., Ye J., Figg W.D., Hwang K., Chung E.J., Murgo A. (2005). Phase I and Pharmacokinetic Study of MS-275, a Histone Deacetylase Inhibitor, in Patients with Advanced and Refractory Solid Tumors or Lymphoma. J. Clin. Oncol..

[65] Pili R., Salumbides B., Zhao M., Altiok S., Qian D., Zwiebel J., Carducci M.A., Rudek M.A. (2012). Phase I Study of the Histone Deacetylase Inhibitor Entinostat in Combination with 13-Cis Retinoic Acid in Patients with Solid Tumours. Br. J. Cancer.

[66] Witta S.E., Jotte R.M., Konduri K., Neubauer M.A., Spira A.I., Ruxer R.L., Varella-Garcia M., Bunn P.A., Hirsch F.R. (2012). Randomized Phase II Trial of Erlotinib with and Without Entinostat in Patients with Advanced Non–Small-Cell Lung Cancer Who Progressed on Prior Chemotherapy. J. Clin. Oncol..

[67] Yardley D.A., Ismail-Khan R.R., Melichar B., Lichinitser M., Munster P.N., Klein P.M., Cruickshank S., Miller K.D., Lee M.J., Trepel J.B. (2013). Randomized Phase II, Double-Blind, Placebo-Controlled Study of Exemestane with or without Entinostat in Postmenopausal Women with Locally Recurrent or Metastatic Estrogen Receptor-Positive Breast Cancer Progressing on Treatment With a Nonsteroidal Aromatase Inhibitor. J. Clin. Oncol..

[68] Gore L., Rothenberg M.L., O’Bryant C.L., Schultz M.K., Sandler A.B., Coffin D., McCoy C., Schott A., Scholz C., Eckhardt S.G. (2008). A Phase I and Pharmacokinetic Study of the Oral Histone Deacetylase Inhibitor, MS-275, in Patients with Refractory Solid Tumors and Lymphomas. Clin. Cancer Res..

[69] Coiffier B., Pro B., Prince H.M., Foss F., Sokol L., Greenwood M., Caballero D., Borchmann P., Morschhauser F., Wilhelm M. (2012). Results From a Pivotal, Open-Label, Phase II Study of Romidepsin in Relapsed or Refractory Peripheral T-Cell Lymphoma After Prior Systemic Therapy. J. Clin. Oncol..

[70] Piekarz R.L., Frye R., Prince H.M., Kirschbaum M.H., Zain J., Allen S.L., Jaffe E.S., Ling A., Turner M., Peer C.J. (2011). Phase 2 Trial of Romidepsin in Patients with Peripheral T-Cell Lymphoma. Blood.

[71] Jones S.F., Infante J.R., Spigel D.R., Peacock N.W., Thompson D.S., Greco F.A., McCulloch W., Burris III H.A. (2012). Phase 1 Results From a Study of Romidepsin in Combination With Gemcitabine in Patients With Advanced Solid Tumors. Cancer Investig..

[72] Shi W., Lawrence Y.R., Choy H., Werner-Wasik M., Andrews D.W., Evans J.J., Judy K.D., Farrell C.J., Moshel Y., Berger A.C. (2014). Vorinostat as a Radiosensitizer for Brain Metastasis: A Phase I Clinical Trial. J. Neurooncol..

[73] Mahalingam D., Mita M., Sarantopoulos J., Wood L., Amaravadi R.K., Davis L.E., Mita A.C., Curiel T.J., Espitia C.M., Nawrocki S.T. (2014). Combined Autophagy and HDAC Inhibition: A Phase I Safety, Tolerability, Pharmacokinetic, and Pharmacodynamic Analysis of Hydroxychloroquine in Combination with the HDAC Inhibitor Vorinostat in Patients with Advanced Solid Tumors. Autophagy.

[74] Gao X., Shen L., Li X., Liu J. (2019). Efficacy and Toxicity of Histone Deacetylase Inhibitors in Relapsed/Refractory Multiple Myeloma: Systematic Review and Meta-analysis of Clinical Trials. Exp. Ther. Med..

[75] Su J.M., Li X.-N., Thompson P., Ou C.-N., Ingle A.M., Russell H., Lau C.C., Adamson P.C., Blaney S.M. (2011). Phase 1 Study of Valproic Acid in Pediatric Patients with Refractory Solid or CNS Tumors: A Children’s Oncology Group Report. Clin. Cancer Res..

[76] Liu J., Yu Y., Kelly J., Sha D., Alhassan A.-B., Yu W., Maletic M.M., Duffy J.L., Klein D.J., Holloway M.K. (2020). Discovery of Highly Selective and Potent HDAC3 Inhibitors Based on a 2-Substituted Benzamide Zinc Binding Group. ACS Med. Chem. Lett..

[77] Bressi J.C., Jennings A.J., Skene R., Wu Y., Melkus R., Jong R.D., O’Connell S., Grimshaw C.E., Navre M., Gangloff A.R. (2010). Exploration of the HDAC2 Foot Pocket: Synthesis and SAR of Substituted N-(2-Aminophenyl)Benzamides. Bioorg. Med. Chem. Lett..

[78] Wagner F.F., Weïwer M., Steinbacher S., Schomburg A., Reinemer P., Gale J.P., Campbell A.J., Fisher S.L., Zhao W.-N., Reis S.A. (2016). Kinetic and Structural Insights into the Binding of Histone Deacetylase 1 and 2 (HDAC1, 2) Inhibitors. Bioorg. Med. Chem..

[79] Li X., Zhang Y., Jiang Y., Wu J., Inks E.S., Chou C.J., Gao S., Hou J., Ding Q., Li J. (2017). Selective HDAC Inhibitors with Potent Oral Activity against Leukemia and Colorectal Cancer: Design, Structure-Activity Relationship and Anti-Tumor Activity Study. Eur. J. Med. Chem..

[80] Tan S., He F., Kong T., Wu J., Liu Z. (2017). Design, Synthesis and Tumor Cell Growth Inhibitory Activity of 3-Nitro-2 H-Cheromene Derivatives as Histone Deacetylaes Inhibitors. Bioorg. Med. Chem..

[81] Chen X., Zhao S., Li H., Wang X., Geng A., Cui H., Lu T., Chen Y., Zhu Y. (2019). Design, Synthesis and Biological Evaluation of Novel Isoindolinone Derivatives as Potent Histone Deacetylase Inhibitors. Eur. J. Med. Chem..

[82] Li X., Inks E.S., Li X., Hou J., Chou C.J., Zhang J., Jiang Y., Zhang Y., Xu W. (2014). Discovery of the First *N* -Hydroxycinnamamide-Based Histone Deacetylase 1/3 Dual Inhibitors with Potent Oral Antitumor Activity. J. Med. Chem..

[83] Wagner F.F., Zhang Y.-L., Fass D.M., Joseph N., Gale J.P., Weïwer M., McCarren P., Fisher S.L., Kaya T., Zhao W.-N. (2015). Kinetically Selective Inhibitors of Histone Deacetylase 2 (HDAC2) as Cognition Enhancers. Chem. Sci..

[84] Nepali K., Chang T.-Y., Lai M.-J., Hsu K.-C., Yen Y., Lin T.E., Lee S.-B., Liou J.-P. (2020). Purine/Purine Isoster Based Scaffolds as New Derivatives of Benzamide Class of HDAC Inhibitors. Eur. J. Med. Chem..

[85] Lai M.-J., Ojha R., Lin M.-H., Liu Y.-M., Lee H.-Y., Lin T.E., Hsu K.-C., Chang C.-Y., Chen M.-C., Nepali K. (2019). 1-Arylsulfonyl Indoline-Benzamides as a New Antitubulin Agents, with Inhibition of Histone Deacetylase. Eur. J. Med. Chem..

[86] Wu W.-C., Liu Y.-M., Lin M.-H., Liao Y.-H., Lai M.-J., Chuang H.-Y., Hung T.-Y., Chen C.-H., Liou J.-P. (2020). Design, Synthesis, and Evaluation of N-Phenyl-4-(2-Phenylsulfonamido)-Benzamides as Microtubule-Targeting Agents in Drug-Resistant Cancer Cells, Displaying HDAC Inhibitory Response. Eur. J. Med. Chem..

[87] Xie R., Yao Y., Tang P., Chen G., Liu X., Yun F., Cheng C., Wu X., Yuan Q. (2017). Design, Synthesis and Biological Evaluation of Novel Hydroxamates and 2-Aminobenzamides as Potent Histone Deacetylase Inhibitors and Antitumor Agents. Eur. J. Med. Chem..

[88] Yun F., Cheng C., Ullah S., He J., Zahi M.R., Yuan Q. (2019). Thioether-Based 2-Aminobenzamide Derivatives: Novel HDAC Inhibitors with Potent in Vitro and in Vivo Antitumor Activity. Eur. J. Med. Chem..

[89] Cheng C., Yun F., He J., Ullah S., Yuan Q. (2019). Design, Synthesis and Biological Evaluation of Novel Thioquinazolinone-Based 2-Aminobenzamide Derivatives as Potent Histone Deacetylase (HDAC) Inhibitors. Eur. J. Med. Chem..

[90] Abdizadeh T., Kalani M.R., Abnous K., Tayarani-Najaran Z., Khashyarmanesh B.Z., Abdizadeh R., Ghodsi R., Hadizadeh F. (2017). Design, Synthesis and Biological Evaluation of Novel Coumarin-Based Benzamides as Potent Histone Deacetylase Inhibitors and Anticancer Agents. Eur. J. Med. Chem..

[91] Wang F., Wang C., Wang J., Zou Y., Chen X., Liu T., Li Y., Zhao Y., Li Y., He B. (2019). N*^ɛ^*-Acetyl Lysine Derivatives with Zinc Binding Groups as Novel HDAC Inhibitors. R. Soc. Open Sci..

[92] Li Y., Wang Y., Xie N., Xu M., Qian P., Zhao Y., Li S. (2015). Design, Synthesis and Antiproliferative Activities of Novel Benzamides Derivatives as HDAC Inhibitors. Eur. J. Med. Chem..

[93] Yu W., Liu J., Clausen D., Yu Y., Duffy J.L., Wang M., Xu S., Deng L., Suzuki T., Chung C.C. (2020). Discovery of Ethyl Ketone-Based Highly Selective HDACs 1, 2, 3 Inhibitors for HIV Latency Reactivation with Minimum Cellular Potency Serum Shift and Reduced HERG Activity. J. Med. Chem..

[94] Hamoud M.M.S., Pulya S., Osman N.A., Bobde Y., Hassan A.E.A., Abdel-Fattah H.A., Ghosh B., Ghanim A.M. (2020). Design, Synthesis, and Biological Evaluation of Novel Nicotinamide Derivatives as Potential Histone Deacetylase-3 Inhibitors. New J. Chem..

[95] Krishna S., Lakra A.D., Shukla N., Khan S., Mishra D.P., Ahmed S., Siddiqi M.I. (2020). Identification of Potential Histone Deacetylase1 (HDAC1) Inhibitors Using Multistep Virtual Screening Approach Including SVM Model, Pharmacophore Modeling, Molecular Docking and Biological Evaluation. J. Biomol. Struct. Dyn..

[96] Farag A.B., Ewida H.A., Ahmed M.S. (2018). Design, Synthesis, and Biological Evaluation of Novel Amide and Hydrazide Based Thioether Analogs Targeting Histone Deacteylase (HDAC) Enzymes. Eur. J. Med. Chem..

[97] Tilekar K., Upadhyay N., Jänsch N., Schweipert M., Mrowka P., Meyer-Almes F.J., Ramaa C.S. (2020). Discovery of 5-Naphthylidene-2,4-Thiazolidinedione Derivatives as Selective HDAC8 Inhibitors and Evaluation of Their Cytotoxic Effects in Leukemic Cell Lines. Bioorg. Chem..

[98] Mohan R., Sharma A.K., Gupta S., Ramaa C.S. (2012). Design, Synthesis, and Biological Evaluation of Novel 2,4-Thiazolidinedione Derivatives as Histone Deacetylase Inhibitors Targeting Liver Cancer Cell Line. Med. Chem. Res..

[99] Upadhyay N., Tilekar K., Jänsch N., Schweipert M., Hess J.D., Henze Macias L., Mrowka P., Aguilera R.J., Choe J., Meyer-Almes F.-J. (2020). Discovery of Novel N-Substituted Thiazolidinediones (TZDs) as HDAC8 Inhibitors: In-Silico Studies, Synthesis, and Biological Evaluation. Bioorg. Chem..

[100] He J., Wang S., Liu X., Lin R., Deng F., Jia Z., Zhang C., Li Z., Zhu H., Tang L. (2020). Synthesis and Biological Evaluation of HDAC Inhibitors With a Novel Zinc Binding Group. Front. Chem..

[101] Bresciani A., Ontoria J.M., Biancofiore I., Cellucci A., Ciammaichella A., Di Marco A., Ferrigno F., Francone A., Malancona S., Monteagudo E. (2019). Improved Selective Class I HDAC and Novel Selective HDAC3 Inhibitors: Beyond Hydroxamic Acids and Benzamides. ACS Med. Chem. Lett..

[102] Whitehead L., Dobler M.R., Radetich B., Zhu Y., Atadja P.W., Claiborne T., Grob J.E., McRiner A., Pancost M.R., Patnaik A. (2011). Human HDAC Isoform Selectivity Achieved via Exploitation of the Acetate Release Channel with Structurally Unique Small Molecule Inhibitors. Bioorg. Med. Chem..

[103] Debnath S., Debnath T., Bhaumik S., Majumdar S., Kalle A.M., Aparna V. (2019). Discovery of Novel Potential Selective HDAC8 Inhibitors by Combine Ligand-Based, Structure-Based Virtual Screening and in-Vitro Biological Evaluation. Sci. Rep..

[104] Greenwood S.O.R., Chan A.W.E., Hansen D.F., Marson C.M. (2020). Potent Non-Hydroxamate Inhibitors of Histone Deacetylase-8: Role and Scope of an Isoindolin-2-Yl Linker with an α-Amino Amide as the Zinc-Binding Unit. Bioorg. Med. Chem. Lett..

[105] Pidugu V.R., Yarla N.S., Pedada S.R., Kalle A.M., Satya A.K. (2016). Design and Synthesis of Novel HDAC8 Inhibitory 2,5-Disubstituted-1,3,4-Oxadiazoles Containing Glycine and Alanine Hybrids with Anti Cancer Activity. Bioorg. Med. Chem..

[106] Valente S., Trisciuoglio D., De Luca T., Nebbioso A., Labella D., Lenoci A., Bigogno C., Dondio G., Miceli M., Brosch G. (2014). 1,3,4-Oxadiazole-Containing Histone Deacetylase Inhibitors: Anticancer Activities in Cancer Cells. J. Med. Chem..

[107] Rajak H., Agarawal A., Parmar P., Thakur B.S., Veerasamy R., Sharma P.C., Kharya M.D. (2011). 2,5-Disubstituted-1,3,4-Oxadiazoles/Thiadiazole as Surface Recognition Moiety: Design and Synthesis of Novel Hydroxamic Acid Based Histone Deacetylase Inhibitors. Bioorg. Med. Chem. Lett..

[108] Kinzel O., Llauger-Bufi L., Pescatore G., Rowley M., Schultz-Fademrecht C., Monteagudo E., Fonsi M., Gonzalez Paz O., Fiore F., Steinkühler C. (2009). Discovery of a Potent Class I Selective Ketone Histone Deacetylase Inhibitor with Antitumor Activity In Vivo and Optimized Pharmacokinetic Properties. J. Med. Chem..

[109] Wang Y., Stowe R.L., Pinello C.E., Tian G., Madoux F., Li D., Zhao L.Y., Li J.-L., Wang Y., Wang Y. (2015). Identification of Histone Deacetylase Inhibitors with Benzoylhydrazide Scaffold That Selectively Inhibit Class I Histone Deacetylases. Chem. Biol..

[110] Li X., Jiang Y., Peterson Y.K., Xu T., Himes R.A., Luo X., Yin G., Inks E.S., Dolloff N., Halene S. (2020). Design of Hydrazide-Bearing HDACIs Based on Panobinostat and Their P53 and FLT3-ITD Dependency in Antileukemia Activity. J. Med. Chem..

[111] Li X., Peterson Y.K., Inks E.S., Himes R.A., Li J., Zhang Y., Kong X., Chou C.J. (2018). Class I HDAC Inhibitors Display Different Antitumor Mechanism in Leukemia and Prostatic Cancer Cells Depending on Their P53 Status. J. Med. Chem..

[112] Goracci L., Deschamps N., Randazzo G.M., Petit C., Dos Santos Passos C., Carrupt P.-A., Simões-Pires C., Nurisso A. (2016). A Rational Approach for the Identification of Non-Hydroxamate HDAC6-Selective Inhibitors. Sci. Rep..

[113] AlSanea M., Gotina L., Mohamed M.F.A., Grace Thomas Parambi D., Anwar H., Mathew B., Youssif B.G.M., Alharbi K.S., Elsayed Z., Abdelgawad M. (2020). Design, Synthesis and Biological Evaluation of New HDAC1 and HDAC2 Inhibitors Endowed with Ligustrazine as a Novel Cap Moiety. Drug Des. Devel. Ther..

[114] McClure J.J., Zhang C., Inks E.S., Peterson Y.K., Li J., Chou C.J. (2016). Development of Allosteric Hydrazide-Containing Class I Histone Deacetylase Inhibitors for Use in Acute Myeloid Leukemia. J. Med. Chem..

[115] Son S.I., Cao J., Zhu C.-L., Miller S.P., Lin H. (2019). Activity-Guided Design of HDAC11-Specific Inhibitors. ACS Chem. Biol..

[116] Madsen A.S., Kristensen H.M.E., Lanz G., Olsen C.A. (2014). The Effect of Various Zinc Binding Groups on Inhibition of Histone Deacetylases 1-11. ChemMedChem.

[117] Liu J., Kelly J., Yu W., Clausen D., Yu Y., Kim H., Duffy J.L., Chung C.C., Myers R.W., Carroll S. (2020). Selective Class I HDAC Inhibitors Based on Aryl Ketone Zinc Binding Induce HIV-1 Protein for Clearance. ACS Med. Chem. Lett..

[118] Veale C.A., Bernstein P.R., Bohnert C.M., Brown F.J., Bryant C., Damewood J.R., Earley R., Feeney S.W., Edwards P.D., Gomes B. (1997). Orally Active Trifluoromethyl Ketone Inhibitors of Human Leukocyte Elastase. J. Med. Chem..

[119] Frey R.R., Wada C.K., Garland R.B., Curtin M.L., Michaelides M.R., Li J., Pease L.J., Glaser K.B., Marcotte P.A., Bouska J.J. (2002). Trifluoromethyl Ketones as Inhibitors of Histone Deacetylase. Bioorg. Med. Chem. Lett..

[120] Scarpelli R., Di Marco A., Ferrigno F., Laufer R., Marcucci I., Muraglia E., Ontoria J.M., Rowley M., Serafini S., Steinkühler C. (2008). Studies of the Metabolic Stability in Cells of 5-(Trifluoroacetyl)Thiophene-2-Carboxamides and Identification of More Stable Class II Histone Deacetylase (HDAC) Inhibitors. Bioorg. Med. Chem. Lett..

[121] Yu W., Liu J., Yu Y., Zhang V., Clausen D., Kelly J., Wolkenberg S., Beshore D., Duffy J.L., Chung C.C. (2020). Discovery of Ethyl Ketone-Based HDACs 1, 2, and 3 Selective Inhibitors for HIV Latency Reactivation. Bioorg. Med. Chem. Lett.

[122] Gong C.-J., Gao A.-H., Zhang Y.-M., Su M.-B., Chen F., Sheng L., Zhou Y.-B., Li J.-Y., Li J., Nan F.-J. (2016). Design, Synthesis and Biological Evaluation of Bisthiazole-Based Trifluoromethyl Ketone Derivatives as Potent HDAC Inhibitors with Improved Cellular Efficacy. Eur. J. Med. Chem..

[123] Schweipert M., Jänsch N., Sugiarto W.O., Meyer-Almes F.-J. (2019). Kinetically Selective and Potent Inhibitors of HDAC8. Biol. Chem..

[124] Bottomley M.J., Lo Surdo P., Di Giovine P., Cirillo A., Scarpelli R., Ferrigno F., Jones P., Neddermann P., De Francesco R., Steinkühler C. (2008). Structural and Functional Analysis of the Human HDAC4 Catalytic Domain Reveals a Regulatory Structural Zinc-Binding Domain. J. Biol. Chem..

[125] Bürli R.W., Luckhurst C.A., Aziz O., Matthews K.L., Yates D., Lyons K.A., Beconi M., McAllister G., Breccia P., Stott A.J. (2013). Design, Synthesis, and Biological Evaluation of Potent and Selective Class IIa Histone Deacetylase (HDAC) Inhibitors as a Potential Therapy for Huntington’s Disease. J. Med. Chem..

[126] Jose B., Oniki Y., Kato T., Nishino N., Sumida Y., Yoshida M. (2004). Novel Histone Deacetylase Inhibitors: Cyclic Tetrapeptide with Trifluoromethyl and Pentafluoroethyl Ketones. Bioorg. Med. Chem. Lett..

[127] Clausen D.J., Liu J., Yu W., Duffy J.L., Chung C.C., Myers R.W., Klein D.J., Fells J., Holloway K., Wu J. (2020). Development of a Selective HDAC Inhibitor Aimed at Reactivating the HIV Latent Reservoir. Bioorg. Med. Chem. Lett..

[128] Traoré M.D.M., Zwick V., Simões-Pires C.A., Nurisso A., Issa M., Cuendet M., Maynadier M., Wein S., Vial H., Jamet H. (2017). Hydroxyl Ketone-Based Histone Deacetylase Inhibitors To Gain Insight into Class I HDAC Selectivity versus that of HDAC6. ACS Omega.

[129] Depetter Y., Geurs S., Vanden Bussche F., De Vreese R., Franceus J., Desmet T., De Wever O., D’hooghe M. (2018). Assessment of the Trifluoromethyl Ketone Functionality as an Alternative Zinc-Binding Group for Selective HDAC6 Inhibition. MedChemComm.

[130] Wouters M.A., Fan S.W., Haworth N.L. (2010). Disulfides as Redox Switches: From Molecular Mechanisms to Functional Significance. Antioxid. Redox Signal..

[131] Nakajima H., Hori Y., Fujita T., Nishimura M., Goto T., Okuhara M. (1994). FR901228, a novel antitumor bicyclic depsipeptide produced by Chromobacterium violaceum No. 968. I. Taxonomy, fermentation, isolation, physico-chemical and biological properties, and antitumor activity. J. Antibiot..

[132] Guan P., Fang H. (2010). Clinical Development of Histone Deacetylase Inhibitor Romidepsin. Drug Discov. Ther..

[133] Yiqiang C., Cheng W. (2011). Histone Deacetylase Inhibitors and Uses Thereof. U.S. Patent.

[134] Biggins J.B., Gleber C.D., Brady S.F. (2011). Acyldepsipeptide HDAC Inhibitor Production Induced in *Burkholderia Thailandensis*. Org. Lett..

[135] Wang C., Flemming C.J., Cheng Y.-Q. (2012). Discovery and Activity Profiling of Thailandepsins A through F, Potent Histone Deacetylase Inhibitors, from Burkholderia Thailandensis E264. MedChemComm.

[136] Cole K.E., Dowling D.P., Boone M.A., Phillips A.J., Christianson D.W. (2011). Structural Basis of the Antiproliferative Activity of Largazole, a Depsipeptide Inhibitor of the Histone Deacetylases. J. Am. Chem. Soc..

[137] Giannini G., Vesci L., Battistuzzi G., Vignola D., Milazzo F.M., Guglielmi M.B., Barbarino M., Santaniello M., Fantò N., Mor M. (2014). ST7612AA1, a Thioacetate-ω(γ-Lactam Carboxamide) Derivative Selected from a Novel Generation of Oral HDAC Inhibitors. J. Med. Chem..

[138] Lv W., Zhang G., Barinka C., Eubanks J.H., Kozikowski A.P. (2017). Design and Synthesis of Mercaptoacetamides as Potent, Selective, and Brain Permeable Histone Deacetylase 6 Inhibitors. ACS Med. Chem. Lett..

[139] Brosowsky J., Lutterbeck M., Liebich A., Keller M., Herp D., Vogelmann A., Jung M., Breit B. (2020). Syntheses of Thailandepsin B Pseudo-Natural Products: Access to New Highly Potent HDAC Inhibitors via Late-Stage Modification. Chem. Eur. J..

[140] Suzuki T., Matsuura A., Kouketsu A., Nakagawa H., Miyata N. (2005). Identification of a Potent Non-Hydroxamate Histone Deacetylase Inhibitor by Mechanism-Based Drug Design. Bioorg. Med. Chem. Lett..

[141] Chen B., Petukhov P.A., Jung M., Velena A., Eliseeva E., Dritschilo A., Kozikowski A.P. (2005). Chemistry and Biology of Mercaptoacetamides as Novel Histone Deacetylase Inhibitors. Bioorg. Med. Chem. Lett..

[142] Porter N.J., Shen S., Barinka C., Kozikowski A.P., Christianson D.W. (2018). Molecular Basis for the Selective Inhibition of Histone Deacetylase 6 by a Mercaptoacetamide Inhibitor. ACS Med. Chem. Lett..

[143] Wen J., Bao Y., Niu Q., Yang J., Fan Y., Li J., Jing Y., Zhao L., Liu D. (2016). Identification of N-(6-Mercaptohexyl)-3-(4-Pyridyl)-1H-Pyrazole-5-Carboxamide and Its Disulfide Prodrug as Potent Histone Deacetylase Inhibitors with In Vitro and In Vivo Anti-Tumor Efficacy. Eur. J. Med. Chem..

[144] Baud M.G.J., Leiser T., Haus P., Samlal S., Wong A.C., Wood R.J., Petrucci V., Gunaratnam M., Hughes S.M., Buluwela L. (2012). Defining the Mechanism of Action and Enzymatic Selectivity of Psammaplin A against Its Epigenetic Targets. J. Med. Chem..

[145] Baud M.G.J., Haus P., Leiser T., Meyer-Almes F.-J., Fuchter M.J. (2013). Highly Ligand Efficient and Selective *N*-2-(Thioethyl)Picolinamide Histone Deacetylase Inhibitors Inspired by the Natural Product Psammaplin A. ChemMedChem.

[146] Gottlicher M. (2001). Valproic Acid Defines a Novel Class of HDAC Inhibitors Inducing Differentiation of Transformed Cells. EMBO J..

[147] Phiel C.J., Zhang F., Huang E.Y., Guenther M.G., Lazar M.A., Klein P.S. (2001). Histone Deacetylase Is a Direct Target of Valproic Acid, a Potent Anticonvulsant, Mood Stabilizer, and Teratogen. J. Biol. Chem..

[148] Lea M.A., Shareef A., Sura M., desBordes C. (2004). Induction of Histone Acetylation and Inhibition of Growth by Phenyl Alkanoic Acids and Structurally Related Molecules. Cancer Chemother. Pharmacol..

[149] Huber K., Doyon G., Plaks J., Fyne E., Mellors J.W., Sluis-Cremer N. (2011). Inhibitors of Histone Deacetylases. J. Biol. Chem..

[150] Newman J.C., Verdin E. (2014). Ketone Bodies as Signaling Metabolites. Trends Endocrinol. Metab..

[151] Chriett S., Dąbek A., Wojtala M., Vidal H., Balcerczyk A., Pirola L. (2019). Prominent Action of Butyrate over β-Hydroxybutyrate as Histone Deacetylase Inhibitor, Transcriptional Modulator and Anti-Inflammatory Molecule. Sci. Rep..

[152] Wang X., Wu X., Liu Q., Kong G., Zhou J., Jiang J., Wu X., Huang Z., Su W., Zhu Q. (2017). Ketogenic Metabolism Inhibits Histone Deacetylase (HDAC) and Reduces Oxidative Stress After Spinal Cord Injury in Rats. Neuroscience.

[153] Lobera M., Madauss K.P., Pohlhaus D.T., Wright Q.G., Trocha M., Schmidt D.R., Baloglu E., Trump R.P., Head M.S., Hofmann G.A. (2013). Selective Class IIa Histone Deacetylase Inhibition via a Nonchelating Zinc-Binding Group. Nat. Chem. Biol..

[154] Guerriero J.L., Sotayo A., Ponichtera H.E., Castrillon J.A., Pourzia A.L., Schad S., Johnson S.F., Carrasco R.D., Lazo S., Bronson R.T. (2017). Class IIa HDAC Inhibition Reduces Breast Tumours and Metastases through Anti-Tumour Macrophages. Nature.

[155] Stott A.J., Maillard M.C., Beaumont V., Allcock D., Aziz O., Borchers A.H., Blackaby W., Breccia P., Creighton-Gutteridge G., Haughan A.F. (2021). Evaluation of 5-(Trifluoromethyl)-1,2,4-Oxadiazole-Based Class IIa HDAC Inhibitors for Huntington’s Disease. ACS Med. Chem. Lett..

[156] Hebach C., Kallen J., Nozulak J., Tintelnot-Blomley M., Widler L. (2013). Novel Trifluoromethyl-Oxadiazole Derivatives and Their Use in the Treatment of Disease.

[157] Lee J., Han Y., Kim Y., Min J., Bae M., Kim D., Jin S., Kyung J. (2017). 1,3,4-Oxadiazole Sulfamide Derivative Compounds as Histone Deacetylase 6 Inhibitor, and the Pharmaceutical Composition Comprising the Same.

[158] Fröhlich E., Wahl R. (2015). Chemotherapy and Chemoprevention by Thiazolidinediones. BioMed Res. Int..

[159] Thuan N.T., Dung D.T.M., Que D.N., Dung P.T.P., Vu T.K., Hahn H., Han B.W., Kim Y., Han S.-B., Nam N.-H. (2015). Synthesis and Bioevaluation of New 5-Benzylidenethiazolidine-2,4-Diones Bearing Benzenesulfonamide Moiety. Med. Chem. Res..

[160] Tilekar K., Hess J.D., Upadhyay N., Bianco A.L., Schweipert M., Laghezza A., Loiodice F., Meyer-Almes F.-J., Aguilera R.J., Lavecchia A. (2021). Thiazolidinedione “Magic Bullets” Simultaneously Targeting PPARγ and HDACs: Design, Synthesis, and Investigations of Their In Vitro and In Vivo Antitumor Effects. J. Med. Chem..

[161] Tilekar K., Hess J.D., Upadhyay N., Schweipert M., Gutierrez D.A., Loiodice F., Lavecchia A., Meyer-Almes F.-J., Aguilera R.J., Ramaa C.S. (2021). Novel Thiazolidinedione (TZD) Derivatives Incorporating Cyclic Linker as HDAC4 Inhibitors: Design, Synthesis and in Vitro Antitumor Evaluation. ChemistrySelect.

[162] Upadhyay N., Tilekar K., Safuan S., Kumar A.P., Schweipert M., Meyer-Almes J., Ramaa C.S. (2021). Multi-Target Weapons: Diaryl-Pyrazoline Thiazolidinediones Simultaneously Targeting VEGFR-2 and HDAC Cancer Hallmarks. RSC Med. Chem..

[163] Kleinschek A., Meyners C., Digiorgio E., Brancolini C., Meyer-Almes F.-J. (2016). Potent and Selective Non-Hydroxamate Histone Deacetylase 8 Inhibitors. ChemMedChem.

[164] Muth M., Jänsch N., Kopranovic A., Krämer A., Wössner N., Jung M., Kirschhöfer F., Brenner-Weiß G., Meyer-Almes F.-J. (2019). Covalent Inhibition of Histone Deacetylase 8 by 3,4-Dihydro-2H-Pyrimido[1,2-c][1,3]Benzothiazin-6-Imine. Biochim. Biophys. Acta Gen. Subj..

[165] Tan W., Jänsch N., Öhlmann T., Meyer-Almes F.-J., Jiang X. (2019). Thiocarbonyl Surrogate via Combination of Potassium Sulfide and Chloroform for Dithiocarbamate Construction. Org. Lett..

[166] Wolff B., Jänsch N., Sugiarto W.O., Frühschulz S., Lang M., Altintas R., Oehme I., Meyer-Almes F.-J. (2019). Synthesis and Structure Activity Relationship of 1, 3-Benzo-Thiazine-2-Thiones as Selective HDAC8 Inhibitors. Eur. J. Med. Chem..

[167] Meyer-Almes F.-J., Meyners C., Kleinschek A., Haus P. (2021). Selective HDAC8 Inhibitors and Their Uses.

[168] Dawood M., Elbadawi M., Böckers M., Bringmann G., Efferth T. (2020). Molecular Docking-Based Virtual Drug Screening Revealing an Oxofluorenyl Benzamide and a Bromonaphthalene Sulfonamido Hydroxybenzoic Acid as HDAC6 Inhibitors with Cytotoxicity against Leukemia Cells. Biomed. Pharmacother..

[169] Pandey M., Kaur P., Shukla S., Abbas A., Fu P., Gupta S. (2012). Plant Flavone Apigenin Inhibits HDAC and Remodels Chromatin to Induce Growth Arrest and Apoptosis in Human Prostate Cancer Cells: In Vitro and In Vivo Study. Mol. Carcinog..

[170] Ononye S.N., VanHeyst M.D., Oblak E.Z., Zhou W., Ammar M., Anderson A.C., Wright D.L. (2013). Tropolones As Lead-Like Natural Products: The Development of Potent and Selective Histone Deacetylase Inhibitors. ACS Med. Chem. Lett..

[171] Haney S.L., Allen C., Varney M.L., Dykstra K.M., Falcone E.R., Colligan S.H., Hu Q., Aldridge A.M., Wright D.L., Wiemer A.J. (2017). Novel Tropolones Induce the Unfolded Protein Response Pathway and Apoptosis in Multiple Myeloma Cells. Oncotarget.

[172] Li Y., Woster P.M. (2015). Discovery of a New Class of Histone Deacetylase Inhibitors with a Novel Zinc Binding Group. MedChemComm.

